# Efficient feature extraction from highly sparse binary genotype data for cancer prognosis prediction using an auto-encoder

**DOI:** 10.3389/fonc.2022.1091767

**Published:** 2023-01-10

**Authors:** Junjie Shen, Huijun Li, Xinghao Yu, Lu Bai, Yongfei Dong, Jianping Cao, Ke Lu, Zaixiang Tang

**Affiliations:** ^1^ Department of Biostatistics, School of Public Health, Medical College of Soochow University, Suzhou, China; ^2^ Jiangsu Key Laboratory of Preventive and Translational Medicine for Geriatric Diseases, Medical College of Soochow University, Suzhou, China; ^3^ Center for Genetic Epidemiology and Genomics, School of Public Health, Medical College of Soochow University, Suzhou, China; ^4^ School of Radiation Medicine and Protection and Collaborative Innovation Center of Radiation Medicine of Jiangsu Higher Education Institutions, Soochow University, Suzhou, China; ^5^ Department of Orthopedics, Affiliated Kunshan Hospital of Jiangsu University, Suzhou, China

**Keywords:** auto-encoder, highly sparse binary data, feature extraction, risk prediction, LASSO

## Abstract

Genomics involving tens of thousands of genes is a complex system determining phenotype. An interesting and vital issue is how to integrate highly sparse genetic genomics data with a mass of minor effects into a prediction model for improving prediction power. We find that the deep learning method can work well to extract features by transforming highly sparse dichotomous data to lower-dimensional continuous data in a non-linear way. This may provide benefits in risk prediction-associated genotype data. We developed a multi-stage strategy to extract information from highly sparse binary genotype data and applied it for cancer prognosis. Specifically, we first reduced the size of binary biomarkers *via* a univariable regression model to a moderate size. Then, a trainable auto-encoder was used to learn compact features from the reduced data. Next, we performed a LASSO problem process to select the optimal combination of extracted features. Lastly, we applied such feature combination to real cancer prognostic models and evaluated the raw predictive effect of the models. The results indicated that these compressed transformation features could better improve the model’s original predictive performance and might avoid an overfitting problem. This idea may be enlightening for everyone involved in cancer research, risk reduction, treatment, and patient care *via* integrating genomics data.

## 1 Introduction

Modern omics technologies can generate large-scale molecular data, such as genomic, transcriptomic, proteomic, and metabolomic data, inducing the opportunity to build more accurate predictive and prognostic models (1, 2). These data have been used to provide tailored healthcare and precision medicine for many individuals (3). However, such data also present computational and statistical challenges because the complexity of the algorithms grows fast with the number of variables.

The underlying representation of many real processes is often sparse. It is of benefit to be able to efficiently eliminate features in a pre-processing step. From the perspective of data dimension reduction, it can be classified into feature selection and feature extraction. Most existing work on feature selection are based on a variant of *l*1-norm penalty due to its sparsity-induced property, strong theoretical guarantees, and great empirical success in kinds of applications (4). The paper about the least absolute shrinkage and selection operator (LASSO) has had an enormous influence (5).

Count data are ubiquitous in genetic risk studies, where it is highly possible to observe excessive zero counts in rare mutation loci. In the face of mass mutation loci, many penalty methods have been adopted in GWAS analyses to select key genetic loci (6–8). For example, Yang et al. detected genetic risk factors among millions of single-nucleotide polymorphisms (SNPs) in ADNI whole genome sequencing data *via* the LASSO method along with the EDPP screening rules (9). Another solution lies in reducing the number of markers before employing a shrinkage method in genetic model such as (10). “Clumping and thresholding” is a two-step method that is often used to derive polygenic risk score (PRS) from results of GWAS studies (11).

Genetic variation is considered associated with cancer prognosis. However, there is little literature on the use of genetic omics data to predict cancer outcomes. As a matter of fact, it is well documented that a large number of genetic markers and generally the small size of their effects make much of the heritability hidden, as a mass of variants with weak effects on disease usually fail to reach the prespecified thresholds of significance (12). It is always an interesting issue how to aggregate these small effects. To better utilize big data in reasoning systems, feature extraction rather than feature selection may allow for discovery of new pathways and principles (13). We identified the auto-encoder as a promising tool. The auto-encoder is a derivative of artificial neural networks (ANNs), with the aim of learning compact and efficient representations from the input data (14). Usually, these representations have a much lower dimension. Departing from supervised ANNs whose performance depends on the quality of gold standards, the auto-encoder directly uses unlabeled data, i.e., the input data itself is the target of reconstruction. Compared to commonly used feature extraction approaches like principal component analysis or independent component analysis that linearly map input to features, the auto-encoder extracts features into non-linear space and work much better as a tool to reduce dimensionality of data (13).

To sum up, we identified that the auto-encoder could learn compact and efficient features from highly sparse binary data and accordingly developed a multiple-stage process to extract information from binary genotype data and applied it for cancer prognosis. In the first stage (screening), we reduced the number of markers *via* a univariable regression model to a moderate size. In the second stage (extracting), we used a trainable auto-encoder to extract representations from the reduced data. In the third stage (selecting), we performed a LASSO process over a grid of tuning parameter values to select the optimal combination of the extracted features. Finally, we applied such feature combination to cancer prognostic models, and evaluated the raw predictive effect of the models.

## 2 Materials and methods

### 2.1 The construction of auto-encoders

A simple auto-encoder is much similar to the ANNs, which generally contains three layers: an input layer, a hidden layer, and a reconstructed layer (output layer) (15). The hidden layer corresponds to the constructed features, with each neuron node representing one feature. The reconstructed layer and the input layer had the same dimensions, and the objective optimized function for the algorithm was to minimize the difference between the two layers.

Let us recall the traditional auto-encoder model proposed by Bengio et al. (16). As many machine learning methods do, we first normalize the continuous input data by the formula (x − x_min_)/(x_max_ − x_min_). Thus, an auto-encoder with “p” features takes an input vector **x** in [0, 1]^p^. The hidden layer representation **y** with “d” dimension is constructed through a deterministic mapping **y** = *f_θ_* (**x**) = *s*(**Wx** + **b**), parameterized by *θ* = {**W**, **b**}. **W** is a “p × d” weight matrix and **b** is a bias vector. Function *s*(x) is called activation function, which introduces nonlinear properties into the network. Common activation functions include (1) rectified linear unit (ReLU) function and (2) sigmoid function:


(1) $$\text{f}\left(\text{x}\right)=\{\begin{array}{c} x,\;x\geq 0\\ 0,\;x<0 \end{array}\;$$

(2) $$\text{g}\left(\text{x}\right)=\frac{1}{1+{\text{e}}^{-\text{x}}}\;$$

Equation (1) maps a linear set of input values to an interval ranging from [0, *∞* ) and equation (2) maps a linear set of input values to an interval in [0, 1]. The value contained in the latent representation **y** for each neuron node is termed the activity value. Then, the resulting hidden layer **y** is mapped back to a “reconstructed” vector **z** in [0, 1]^p^ in a similar manner, by inputting space **z** = *g_θ’_* (**y**) = *h*(**W’y** + **b’**) with *θ’* = {**W’**, **b’**}. The function *h*(x) is also an activation function, restoring the latent information to the original information. We could use tied weights if the two activation functions are the same, which means that the transpose of **W** was used for **W’**. The parameters in this neural network are optimized to minimize the average reconstruction loss between the input layer **x** and the reconstructed layer **z**:


(3) $$\theta ,\;\theta^{'}{=}_{\;\theta ,\;\theta '\;}^{\text{arg min}}1/\text{n}\overset{\text{n}}{\underset{\text{i}=1}{\sum }}L(x^{\left(i\right)}{\text{,z}}^{\left(i\right)}\text{) }$$

where n is the sample size and *L* is a loss function like squared error loss function *L*(**x**, **z**) = ||**x**−**z**||^2^. An alternative error loss, cross-entropy loss function, is suggested by the interpretation of **x** and **z** as vectors of bit probabilities:


(4) $$L_{H}\left(x,\;z\right)=-\sum_{\text{k}=1}^{\text{p}}\left[x_{\text{k}}\text{log }z_{\text{k}}+\left(1-x_{\text{k}}\right)\text{log}\left(1-z_{\text{k}}\right)\right]\;$$

Like other feed-forward ANNs, the auto-encoder takes back propagation algorithm and gradient descent algorithm to compute and update target parameters iteratively until reaching an acceptable loss or the given epochs. The specific theory can be referred to the relevant literature (17).

### 2.2 The LASSO and its selection rules

Given a linear regression with standardized predictors x_ij_ and centered response values y_i_ for i = 1, 2,…, N (samples) and j = 1, 2,…, p (features), the LASSO solves the *l*1-penalized regression problem for finding *β* = {*β_j_*} to minimize


(5) $$\sum_{i=1}^{N}{\left(y_{i}-\underset{j}{\sum }x_{ij}\beta_{j}\right)}^{2}+\lambda \sum_{j=1}^{p}\left|\beta_{j}\right|\;$$

where λ ≥ 0 is a tuning parameter.

A main reason for using the LASSO is that the *l*1-penalty tends to set some entries of $\tilde{\beta }$ to 0, and therefore, it performs a kind of variable selection. Furthermore, Tibshirani (18) proposed “strong rules” to discard noise signal in the LASSO-type penalty problems. The results indicated that the LASSO performs well in both low signal-to-noise ratio (SNR) and high sparse regimes by incorporating the “strong rules”. However, the predictor matrices from their simulated studies were all generated from Gaussian distribution. Subsequent simulation studies that aimed to improve variable selection algorithm using a LASSO-type penalty still concerned continuous predictors mainly (19–21). Guo et al. considered the power of the LASSO for SNP selection in predicting quantitative traits and proved that the LASSO still has good selection ability for high-dimensional and sparse binary predictors (22). However, when the values of these binary predictors become highly sparse (rare mutation) such as 99.9% of zeros and 0.01% of ones, we observed that the power of the LASSO to select non-zero variables declined. This is briefly illustrated in supplementary file part II and **Table S1**.

### 2.3 The property of the auto-encoder to feature selection

We explore the feature extraction capability of the auto-encoder using two visualized image datasets from the Mixed National Institute of Standards and Technology database (MNIST) (23) and fashion MNIST. The MNIST is one of the most widely used benchmark dataset for isolated handwritten digit recognition from 0 to 9. Digits are transformed to 28×28 images, and represented as 784×1 vectors. Each component is a number between 0 and 255, which means the gray levels of each pixel. The number of zeros accounts for about 81%. It has a training set of 60,000 examples, and a test set of 10,000 examples. The fashion MNIST is a substitute for the MNIST dataset and is more complex, consisting of 10 types of wearing images. The number of 0 accounts for about 51%. The above datasets are loaded and accessed through the “Keras” module of TensorFlow. The deep learning framework of the auto-encoder is constructed by the TensorFlow library (2.3.0) of Python (3.7) in the Jupyter Notebook platform (6.3.0).

#### 2.3.1 Handwritten digit recognition

We took the first 1,000 examples of training set as training data and the first 1,000 examples of test set as testing data from the MNIST to study the property of our auto-encoder. First, as mentioned above, we reshaped the 28×28 images to 784×1 vectors and normalized the input data from [0, 255] to [0, 1]. Thus, the dimension of input layer as well as reconstructed layer was 784. We set the hidden layer dimension to 100 (this number is optional). See construction of the auto-encoder in **Figure S1**. Activation function *s*(x) was specified to the ReLU function due to its good property and therefore the activity values in the hidden layer **y** ranging from [0, *∞* ). The activation function *h*(x) could be either ReLU function or sigmoid function, corresponding to mean squared error (MSE) loss and mean cross-entropy (MCE) loss. We used the two activation functions respectively and compared the fitting effects.

In terms of configuration training method, we used the “Adam” optimizer from the “Keras” module. The size of each update is controlled by learning rate. To speed up the training, samples were randomly grouped into batches, and the number of samples contained in a batch was termed the batch size, with weight and bias being updated after each batch. Training proceeded through epochs, and samples were re-batched at the beginning of each epoch. Training was stopped after a specified number of epochs (termed epoch size) was reached. We performed a full factorial design over all combinations of the following parameters: a learning rate of 0.001, 0.005, and 0.010; a batch size of 32, 64, and 128; and an epoch size of 50, 100, and 150. After a full factorial parameter sweep, the parameters that we selected were as follows: a learning rate of 0.005, a batch size of 128, and an epoch size of 100, which could achieve fast training speed and smooth loss.

When using the sigmoid function as activation function *h*(x), the MCE was 0.0683 with a binary accuracy (calculates how often predictions matches labels) of 0.8156 in the training data (see **Figure S2A**) and 0.0898 MCE with 0.8244 accuracy in the testing data using the model built in training data. We read the first five images of the training data and testing data, as shown in **Figures S3A, B**. The first row shows the original images, the second row shows the extracted features, and the third row shows that the images were restored accurately with the extracted features. The results show that the model can be used to extract the key features well. Meanwhile, we used the reconstructed data for handwritten digit prediction and found that the probability of predicting the correct classification was close to 1 (see **Table S2**).

While using the ReLU function as activation function *h*(x), the MSE was 0.0067 with an accuracy (calculates how often predictions matches labels) of 0.0150 in the training data (see **Figure S2B**) and 0.0125 MSE with an accuracy 0.0200 in the testing data using the same model. We also read the first five images of the training data and testing data (**Figures S3C, D**). It shows that the ReLU function performed quite poorer compared to sigmoid function. Because the labels of corresponding output data are normalized data ranging from [0, 1], sigmoid function could work more suitably.

#### 2.3.2 Fashion image recognition

We took the same procedure as *Section 2.3.1* in fashion MNIST data. We selected the first 1,000 examples of training set as training data. The activation function *h*(x) was directly specified to sigmoid function. We set the same configuration training method except for an epoch size of 200. The MCE was 0.2667 with a binary accuracy of 0.5166 in the training data (see **Figure S4A**). We read the first six images of the training data, as shown in **Figure S5A**. We found that the fitting effect was poorer in the fashion MNIST data than in the MNIST data, because the proportion of zeros is lower in the fashion MNIST data (about 51%) than the MNIST data (about 81%).

Inspired by denoising auto-encoders (24), we artificially added some corruption to training data. Specifically, we set values below 0.21 to zeros in the input data, making the proportion of zeros up to about 58.5%. Then, we retrained the model; the MCE was 0.2440 with an accuracy of 0.5924 in the new (corrupted) training data (see **Figure S4B**). The first six images of the new training data are shown in **Figure S5B**. The black icon became a little clearer (e.g., the second on the left, the first on the right). Images before and after the corruption are shown in **Figure S5C**. The first and third images were before the corruption, and the second and fourth images were after the corruption. Our results show that the higher the proportion of 0 and 1, the better the feature extraction effect of the auto-encoder using the sigmoid function.

#### 2.3.3 Auto-encoder feature selection for highly sparse binary predictors

We used the auto-encoder to extract features from the highly sparse binary data. We randomly used simulation data generated from scenario 5 in **Table S1**. The sample size was 200 with 400 binary predictors. Thus, in the testing auto-coder, the dimension of the input layer as well as the reconstructed layer was 400. We set the hidden layer dimension to 100, i.e., extracting 100 important features. We used the “Adam” optimizer, and the parameters that we selected were as follows: a learning rate of 0.005, a batch size of 32, and an epoch size of 200. The activation function *h*(x) was set to sigmoid function.

As a result, the MCE was 0.0001 with a binary accuracy of 1.0000 (see **Figure S6A**). We read the first five “images” of this simulated data, as shown in **Figure S6B**. The auto-encoder could recover the scattered genetic signals and when there was no genetic signal in the sample, an identical noise signal was given. The extracted 100 signal features were then used in LASSO Cox regression, and 9 features were selected. We calculated Harrell’s concordance index (C-index) with 0.670 (standard error, SE = 0.035) and the *R* ^2^ was 0.215. If the LASSO Cox regression were applied directly using 400 binary predictors, a total of 65 predictors were selected (of which 5 were real nonzero predictors). The C-index was 0.721 (SE = 0.030) and the *R* ^2^ was 0.379. The result obtained using the auto-encoder was much more close to the performance of scenario 1 in **Table S1** (average C-index: 0.647, average *R* ^2^: 0.244). Due to the selection of more noise predictors, using the LASSO directly had a virtual height of C-index and *R* ^2^ that would induce overfitting.

## 3 Cancer prognosis application

The Cancer Genome Atlas (TCGA) project was started in 2006 by the National Cancer Institute (NCI) and the National Human Genome Research Institute (NHGRI). The database has contained a variety of cancer data from more than 20,000 samples of 33 types of cancer, including transcriptome expression data, genomic variation data, methylation data, and clinical data. As the largest cancer gene database, TCGA has become the first choice for cancer research due to its large sample size, diverse data types and standardized data formats.

We downloaded the latest (in July 2022) single-nucleotide variation (SNV) data and phenotype data of the GDC TCGA Breast Cancer (BRCA) cohort (female) and GDC TCGA Ovary Cancer (OV) cohort from the official website “GDC Data Portal”. A total of 977 SNV documents and 1,085 phenotype documents were obtained from BRCA and 480 SNV documents and 597 phenotype documents were obtained from OV. The data type of SNV is masked somatic mutation, read and collated by R package *mafTools*. The overview of SNV in BRCA and OV is shown in **Figure S7**. We eliminated data with variants that were nonsense mutation. Next, we used the R package *reshape2* to reshape the mutation data to count how many SNV mutations were present in each gene per patient. Zero means wild type, and one means mutated (genotype data with 0/1 values). The interested phenotype in this study was overall survival (OS).

### 3.1 BRCA data

There were a total of 66,780 SNV items in which 4,910 were nonsense mutation. Many genes had more than one mutation, but we deemed all of them as “mutated” and were labeled “1”. A total of 952 BRCA patients with 15,124 genotype data were available. After merging survival data, those with missing survival data were eliminated and 939 subjects were left. Univariable Cox analysis was performed on these 15,124 genotype data as preliminary screening to identify potential contributors, and 1,936 of them with a *p-*value of less than 0.05 (a rough threshold) were selected for subsequent analysis. We found that if the LASSO Cox regression were applied directly to these 1,936 genotype data, no variables would be selected by LASSO (see **Figure S8**). This was possible in such a scenario because the proportion of zeros reaches 99.6%. Thus, we thought of using an auto-coder to extract features from these highly sparse binary variables. We also consider random survival forest (RSF) as an alternative to screen the key variables because the random forest method is employed to detect significant SNPs in large-scale GWAS (25).

#### 3.1.1 Feature extraction using an auto-encoder and the development of the prognosis model

**Figure 1:**
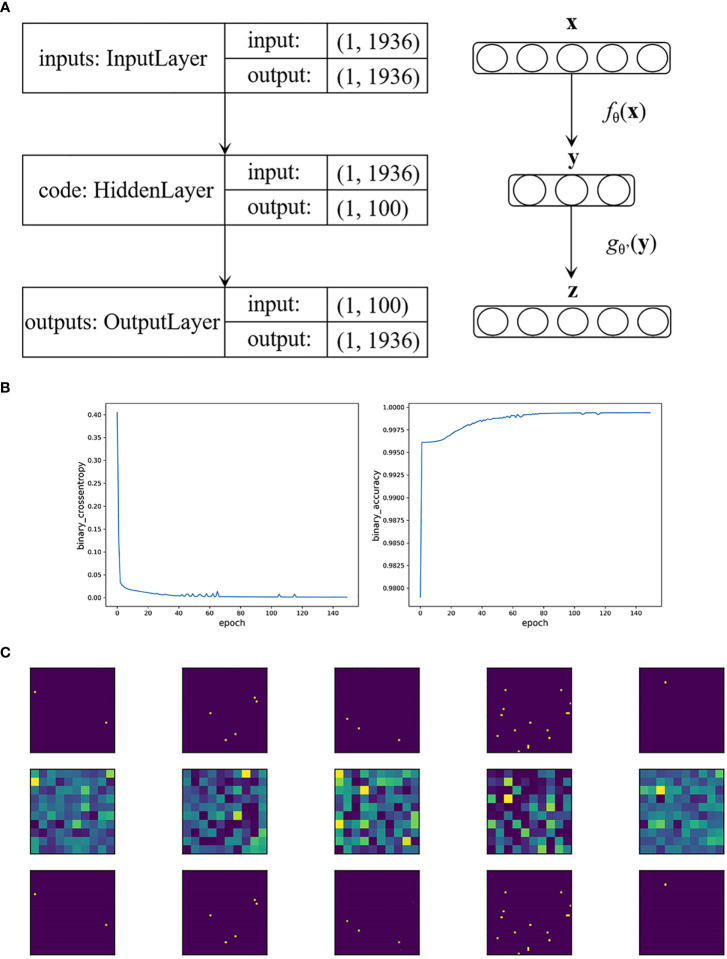
**(A)** The construction of the auto-encoder in BRCA data. **(B)** Loss function value and accuracy of the auto-encoder in BRCA data by the epoch times. **(C)** The first five visualized genetic signal of BRCA data. The first row shows the original images, the second row shows the extracted features, and the third row shows that the images were restored accurately with the extracted features.

Specifically, in our BRCA auto-encoder, the dimension of the input layer as well as the reconstructed layer was 1,936. We set the hidden layer dimension to 100, i.e., extracting 100 important features. **Figure 1A** shows the construction of the auto-encoder. We used the “Adam” optimizer; the parameters that we selected were as follows: a learning rate of 0.005, a batch size of 32, and an epoch size of 150. The activation function *h*(x) was set to sigmoid function with MCE loss.

**Figure 2:**
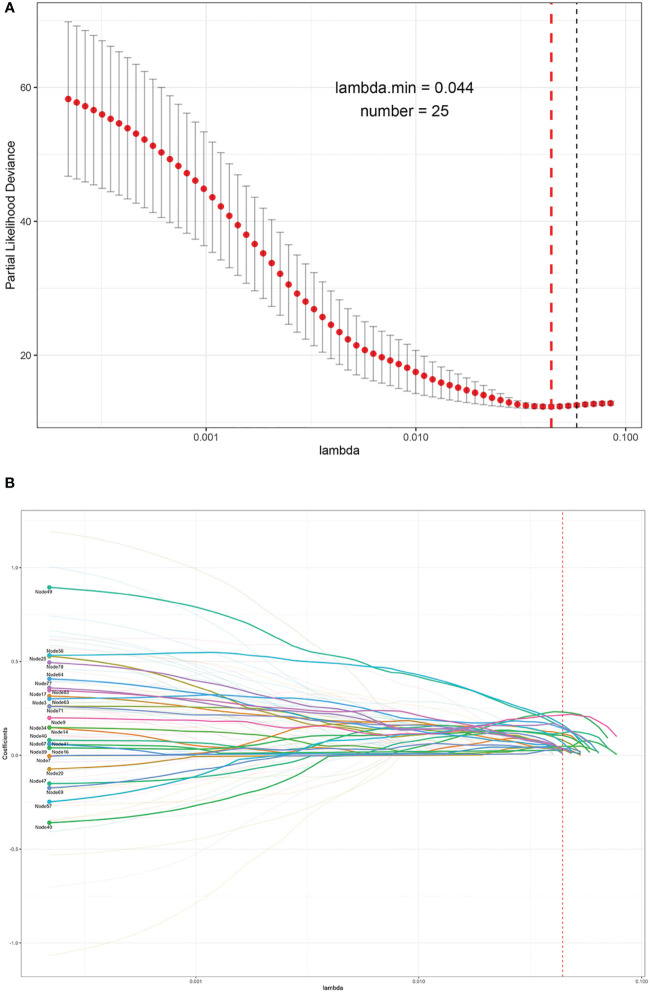
The process of the LASSO to select optimal predictors in BRCA data. **(A)** Penalty parameter tuning conducted by 10-fold cross-validation. **(B)** The solution pathway of the 25 features.

As a result, the MCE was 0.0006 with a binary accuracy of 1.0000 (**Figure 1B**). We read the first five “images” of these data, as shown in **Figure 1C**. The auto-encoder could recover the scattered genetic signals well as expected. The extracted 100 signal features were continuous variables (see **Table S3** for example) and then thrown into the LASSO Cox regression. Finally, 25 features were selected (see **Figure 2**). We build a prognosis signature called SNV signature based on these 25 features using the R functions “predict()”, “cph()”, and “coxph()” among BRCA patients. The mean C-index of this signature was 0.830 (SE = 0.069), and the mean *R* ^2^ was 0.245, which was performed with a fivefold cross-validation process and stepAIC to avoid overfitting.

We used this signature to divide the population into two groups. The optimal cutoff value of the signature was determined using the R package *survminer*. The R package *survival* was used to perform survival analysis between these two groups. The Kaplan–Meier (K-M) curve was used to show difference of survival curves between groups (discrimination). Log-rank test was used to evaluate statistical differences of the survival. The receiver operating characteristic (ROC) curve and its area under the curve (AUC) values were utilized to evaluate the specificity and sensitivity of the signature in a time-dependent manner using the R package *timeROC*. We drew observed survival curves and predicted survival curves to compare the agreement (calibration), by calculating baseline hazard using the R function “basehaz()”. We also assessed calibration with calibration plots. A 45°C diagonal line represents perfect calibration, while deviation below or above this line implies overestimation or underestimation of survival.

**Figure 3:**
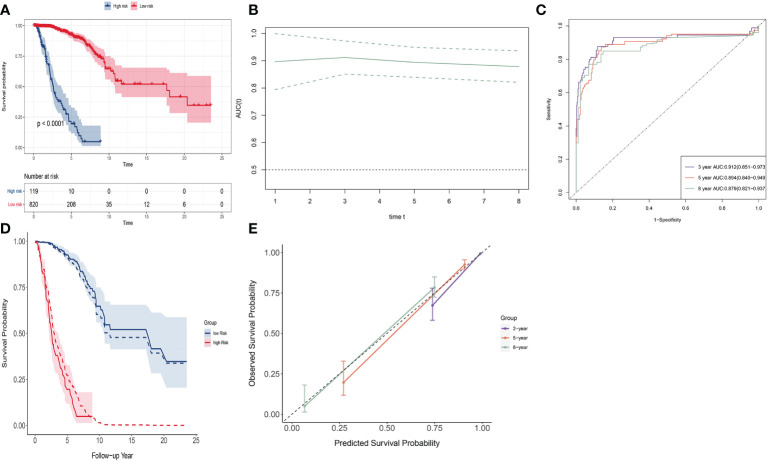
Discrimination and calibration of SNV signature in BRCA data. **(A)** The K-M curve of the low-risk group and the high-risk group. **(B)** Time-dependent AUC of SNV signature. **(C)** The 2-, 5-, and 8-year AUC of SNV signature. **(D)** The observed survival curves (solid line) and predicted survival curves (dotted line). **(E)** Calibration plot for 2-, 5-, and 8-year AUC of SNV signature.

SNV signature ranging from (−3.564, 6.445) with a mean of 0. Patients were divided into a low-risk group (*n* = 820) and a high-risk group (*n* = 119); optimal cutoff value was 1.243 (see **Figure 3A**). The low-risk group had a much higher survival rate compared to the high-risk group (*p* < 0.0001). The 8-year survival rate of the low-risk group was over 0.75, whereas that of the high-risk group was almost 0. The time-dependent AUC curve was approximately 0.9 during 8 years (**Figure 3B**). The 2-, 5-, and 8-year AUC of the signature were 0.928 (95% CI: 0.870–0.987), 0.894 (95% CI: 0.840–0.949), and 0.879 (95% CI: 0.821-0.937), respectively. (**Figure 3C**). The observed survival curves (solid line) and predicted survival curves (dotted line) are shown in **Figure 3D**. The predicted survival curves were in the credible interval. The signature overestimated survival probability for the low-risk group and underestimated survival probability for the high-risk group. The calibration plot of these two groups shows the same result at 2, 5, and 8 years (**Figure 3E**).

**Figure 4:**
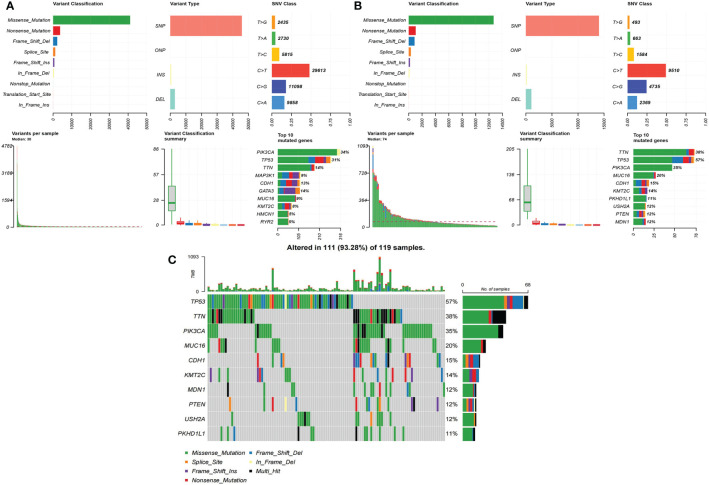
The summary of SNVs in two groups in BRCA data. **(A)** Low-risk group. **(B)** High-risk group. **(C)** The detailed distribution of the top 10 mutated genes in the high-risk group.

For a summary of SNVs in both the low-risk group (**Figure 4A**) and the high-risk group, see **Figure 4B**. The median of variants per sample in the low-risk group was 30 but 74 in the high-risk group. The rank and distribution of the top 10 mutated genes in the low-risk group was similar to the whole population (**Figure S7A**). Peculiarly, we plotted the detailed distribution of the top 10 mutated genes in the high-risk group (**Figure 4C**). Fifty-seven percent of the samples had TP53 mutation in the high-risk group compared to 31% in the low-risk group; 38% of the samples had TTN mutation in the high-risk group compared to 14% in the low-risk group.

#### 3.1.2 RSF for variable screening

RSF is used for prediction and variable selection for right-censored survival and competing risk data (26). A random forest of survival trees is used for ensemble estimation of cumulative hazard function in right-censored settings. Different survival tree splitting rules are used to grow trees. An estimate of C-index is provided for assessing prediction accuracy. Variable importance for single or grouped variables can be used to filter variables and to assess variable predictiveness.

We used the R package *randomSurvivalForest* to build an RSF model and ranked the importance of variables. Number of trees to grow was set to 10,000 in order to ensure that every input row got predicted at least a few times. The result of the model is shown in the **Figure S9**. Prediction error is measured by the 1 − C-index. The estimate of prediction error rate of this model was 0.449 (**Figure S9A**). We selected variables with an importance index greater than 0.3 (21 mutant genes) and plotted them in **Figure S9B**. However, we selected the 100 most important variables (see **Table S4**) and threw them into the LASSO Cox regression model. Twenty-three predictors were left (**Figure S10**). They offered 0.624 (SD = 0.048) of mean C-index and 0.081 of mean *R* ^2^ performed with a fivefold cross-validation process and stepAIC. It was not surprising that the C-index and *R* ^2^ were much lower using the RSF model when compared to using the auto-encoder (they used a similar number of variables: 25 versus 23) because the RSF model only selected the 100 most important variables and the auto-encoder used whole information.

#### 3.1.3 Genotype and gene expression

We also performed univariable Cox analysis with gene expression data of BRCA. Data category is transcriptome profiling, data type is gene expression quantification, and workflow type is “STAR-Counts”. We also selected 1,936 of them with the lowest *p-*value in univariable Cox analysis. Then, the multivariable LASSO Cox was used to select final predictors. A total of 60 predictors were left (**Figure S11**). They offered 0.831 (SD = 0.059) of mean C-index and 0.239 of mean *R* ^2^ performed with a fivefold cross-validation process and stepAIC. We drew a Venn plot of approximately 1,936 genotypes, 1,936 genes, and 60 predictors (see **Figure S12**), and found many common genes. Based on an explicit assumption of temporal ordering from genotype, gene expression, and survival outcome, survival mediation analysis of gene expression with multiple genotype exposures is feasible, referring to (27).

### 3.2 OV data

There were a total of 30,210 SNV items in which 1,650 were nonsense mutation. A total of 406 OV patients with 11,322 genotype data were available. After merging survival data, those with missing survival data were eliminated and 359 subjects were left. Univariable Cox analysis was performed on these 11,322 genotype data, and 1,089 of them with a *p-*value of less than 0.05 were selected for subsequent analysis. Then, the LASSO Cox regression was applied directly to these data, and a total of 95 predictors were selected by LASSO (see **Figure S13A**). The mean C-index was 0.707 (SD = 0.032) and the mean *R* ^2^ was 0.091 performed with a fivefold cross-validation process and stepAIC. We also used the auto-coder to extract features from the 1,089 binary variables. A total of 19 features were selected from 100 extracted features using the LASSO process (see **Figure S13B**). The mean C-index of the 19 features was 0.734 (SD = 0.025) and mean *R* ^2^ was 0.297 performed with a fivefold cross-validation process and stepAIC.

## 4 Discussion

The use of transcriptome data to construct cancer prognostic models has become very popular, and its performance in the internal verification is often satisfactory. However, due to different sequencing platforms and sequencing methods, instability of transcriptome data expression, and data standardization problems, extrapolation is still questionable. Trying to get the same desirable results from a random external data is always going to be less than expected.

SNV is a widely studied type of gene mutation (SNP is the most common type), which exists stably in somatic cells and plays a key role in regulating transcriptome expression. Aggregating small effects of SNV is a convincing attempt with promising applications. Our research shows that auto-encoders can extract effective information from dichotomous data well, even in the case of highly sparse variable values. It maps the linear combination of input dichotomous variables to a continuous value space with a lower dimension by neural networks and activation function. These features can retain most of the original information without worrying about overfitting issues, because our goal is to get the original information as possible. In addition, compared to highly sparse binary variables, low-dimensional continuous variables are better utilized. Therefore, we thought of using the auto-encoder to integrate such highly sparse binary SNV data.

Studies have shown that inherited genetic variation is associated with cancer prognosis (28–30). However, few studies have used SNV information to predict cancer prognosis in female patients. A study using multi-omics data [including gene expression data, copy number variation (CNV) data, and SNP] to predict the prognosis of BRCA patients had a 5-year survival AUC of 0.65 through their six-gene signature (31). By contrast, our study shows the power of feature extraction using the deep learning method. Based on the aggregated SNV information, we can greatly improve the ability to predict cancer patients’ outcome.

In our study, BRCA patients were stratified into a low-risk group and a high-risk group based on the SNV signature. The high-risk group had higher TP53 and TTN mutation. TP53 is a well-known mutated gene and is a mutant in 30% of all breast cancers. It is clear that the role of TP53 in the management of breast cancer matters (32). Moreover, we searched the existing mutational signatures of BRCA in COSMIC (the catalogue of somatic mutations in cancer, https://cancer.sanger.ac.uk/signatures/) and found that TP53 mutation is validated to be concordant with transcriptome expression (33). TTN-AS1 is a long noncoding RNA (lncRNA) that binds to titin mRNA (TTN). Many studies have shown that overexpression of TTN-AS1 correlates with poor prognosis in breast cancer and with more advanced pathology (34).

Furthermore, we searched for studies on SNP analysis with the auto-encoder in PubMed (8, 35–37). The most cutting-edge methods take auto-encoders to extract features from SNP data too (35). Specifically, the authors applied a deep canonically correlated sparse auto-encoder to extract key features from SNP data and functional magnetic resonance imaging (fMRI) data and then stacked these features together for classification. Their approach is very interesting and engaging because they addressed the nonlinear dimension reduction and considered the correlation between the above two types of data. The AUC score of their proposed model for the SNP data was 0.984 and that for fMRI data was 0.953, which were the highest AUC scores among all models. The difference of our study is that we have made an interesting experiment on the feature extraction property of auto-encoders. We compared the selection of activation functions in the output layer and found that the sigmoid function was more suitable for feature extraction than the ReLU function. The effect of dichotomous data was better than continuous data. In addition, the data involved in our study were from publicly available databases; thus, all results are reliable and reproducible.

Our study has its limitations. First, a person’s entire sequencing genome data are not easy to come by, which makes it difficult to verify the performance of the prediction model externally, but it is hoped to be achieved in the future. Second, although we considered the correlation between covariates within and between groups in our simulation study in supplementary files, we did not incorporate genetic elements such as linkage disequilibrium. Third, due to the randomness of parameter initialization, results of deep neural network training are also random. Therefore, the characteristics obtained from each training time are always different. For example, in the BRCA dataset, each time the auto-encoder was retrained, the obtained features used for the LASSO analysis were different, as well as the C-index. However, the difference was not apparent, only causing the raw C-index to move around an interval, say 0.865 to 0.915 (see **Table S5**). Therefore, any training result is feasible in a single test. Furthermore, there may be many other scenarios where deep neural networks can be used to extract features and make use of them. This remains to be discovered by the scholars.

## 5 Conclusion

Integrating minor effects from highly sparse genetic genome data could improve prediction power. We studied the feature extraction property of the auto-encoder and found that it can work well to extract features by transforming highly sparse binary data (e.g., rare mutation) to lower-dimensional continuous data in a non-linear way. We applied this method to two cancer prognosis studies that had genotype data and achieved good predictive performance. This idea may provide something for everyone involved in cancer research, risk reduction, treatment, and patient care.

## Data availability statement

We obtained image data information from MNIST (http://yann.lecun.com/exdb/mnist/) and fashion MNIST (https://jobs.zalando.com/en/tech/?gh_src=281f2ef41us). We obtained data information of BRCA and OV from official website “GDC Data Portal” (https://portal.gdc.cancer.gov/repository).

## Author contributions

Study conception and design: JS, KL, and ZT. Data collection and cleaning: JS and HL. Real data analysis and interpretation: JS, XY, and JC. Drafting of the manuscript: JS, LB, and YD. All authors reviewed the manuscript. All authors contributed to the article and approved the submitted version.
