# Supplementary material for: Efficient feature extraction from highly sparse binary genotype data for cancer prognosis prediction using an auto-encoder
Source: Front Oncol. 2023 Jan 10;12:1091767. doi: 10.3389/fonc.2022.1091767 (PMC9872139; doi:10.3389/fonc.2022.1091767)
Supplement: Supplementary file 1 [file DataSheet_1.docx]

CONTENT

**Part I: Supplementary tables and figures**

[Supplementary Table 1. The performance of the LASSO in different scenarios 1](#_Toc72662154)

[Supplementary Table 2. The predicted probability distribution of the true label using the extracted features 2](#_Toc72662155)

[Supplementary Table 3. The original input data and the extracted features 3](#_Toc72662156)

[Supplementary Table 4. The ranking of the 100 most important variables using RSF 4](#_Toc72662157)

[Supplementary Table 5. The influence of randomness of neural network training on results 5](#_Toc72662152)

[Supplementary Figure 1. The construction of the auto-encoder in MNIST data 6](#_Toc72662160)

[Supplementary Figure 2. Loss function value and accuracy of the auto-encoder in MNIST training data by the epoch times 7](#_Toc72662161)

[Supplementary Figure 3. The first five image of MNIST training data and testing data 11](#_Toc72662162)

[Supplementary Figure 4. Loss function value and accuracy of the auto-encoder in fashion MNIST training data by the epoch times 13](#_Toc72662163)

[Supplementary Figure 5. The first five image of fashion MNIST training data 1](#_Toc72662164)6

[Supplementary Figure 6. Auto-encoder feature selection for highly sparse binary predictors 1](#_Toc72662165)8

[Supplementary Figure 7. The summary of SNVs in BRCA data and OV data 2](#_Toc72662166)0

[Supplementary Figure 8. The process of the LASSO to directly select predictors using 1,936 genotype data in BRCA 2](#_Toc72662167)1

[Supplementary Figure 9. The process of variables selection using RSF 2](#_Toc72662167)3

[Supplementary Figure 10. The process of the LASSO to select predictors using 100 most important variables selected using RSF in BRCA 24](#_Toc72662167)

[Supplementary Figure 11. The process of the LASSO to directly select predictors using 1,936 gene expression data in BRCA 25](#_Toc72662167)

[Supplementary Figure 12. The Venn plot about 1,936 genotype, 1,936 genes and 60 predictors 26](#_Toc72662167)

[Supplementary Figure 13. The process of the LASSO to select predictors using genotype data in OV 28](#_Toc72662167)

**Part II: The LASSO selection for highly sparse binary predictors**

# Supplementary Table 1. The performance of the LASSO in different scenarios.

| scenario | n | nonzero coefficients | m | n.sim | average C index (= sum/100) | average R square (= sum/100) | average Dxy (= sum/100) | average number of nonzero coefficients caught by the LASSO | average number of zero coefficients caught by the LASSO | average total number of variables caught by the LASSO | times of no variables caught by the LASSO |
| --- | --- | --- | --- | --- | --- | --- | --- | --- | --- | --- | --- |
| 1 | 200 | 15 | 15 | 100 | 0.647 | 0.244 | 0.299 | 10.83 | 0 | 10.83 | 2/100 |
| 2 | 200 | 15 | 100 | 100 | 0.659 | 0.249 | 0.318 | 6.11 | 7.59 | 13.7 | 5/100 |
| 3 | 200 | 15 | 200 | 100 | 0.660 | 0.258 | 0.320 | 4.58 | 10.91 | 15.49 | 14/100 |
| 4 | 200 | 15 | 300 | 100 | 0.643 | 0.242 | 0.287 | 3.57 | 10.54 | 14.11 | 19/100 |
| 5 | 200 | 15 | 400 | 100 | 0.629 | 0.219 | 0.257 | 2.96 | 10.19 | 13.15 | 23/100 |

Note: n is sample size; m is number of variables; n.sim is the number of simulation times.

Note: Scenario1 could be seemed as a true performance of the LASSO. As noise variables increase, power of the LASSO to select non-zero coefficients plummets (from 10.83 to 2.96). In addition, the possibility that the LASSO would not be able to pick any predictors increases (from 0.02 to 0.23). Although average C index and average R square were higher in Scenario2 and Scenario3 than Scenario1, the reason was that the LASSO selected much more false variables.

# Supplementary Table 2. The predicted probability distribution of the true label using the extracted features.

| Predicted probability of 0-9 handwritten digit | True label |
| --- | --- |
| [[1.7158746e-07 2.1812367e-09 1.4064822e-08 1.0445129e-05 1.6168548e-19 **9.9998939e-01** 2.3555690e-14 1.1475397e-14 9.7253883e-10 5.5351227e-15] | 5 |
| [**1.0000000e+00** 1.0555389e-13 6.0633349e-14 1.3680860e-13 2.8062880e-18 7.2386666e-11 7.8288166e-12 1.4637241e-10 1.3177336e-15 7.7014062e-11] | 0 |
| [5.1418975e-10 1.6384566e-07 7.2677189e-09 2.6164489e-07 **9.9998760e-01** 1.2450415e-07 1.3080398e-06 2.2331892e-09 2.9598075e-07 1.0230306e-05] | 4 |
| [2.7635500e-13 **9.9997449e-01** 1.6632292e-05 1.7858354e-12 2.8408922e-15 1.6070815e-15 1.7656064e-11 1.5948520e-11 8.8462084e-06 4.6029258e-16] | 1 |
| [3.2652074e-13 1.5590743e-09 4.6157786e-07 1.2088082e-09 7.9517654e-04 4.1053871e-14 6.2286819e-17 5.7289385e-06 1.3418399e-08 **9.9919862e-01**]] | 9 |

# Supplementary Table 3. The original input data and the extracted features.

| The original input data | The extracted features |
| --- | --- |
| KATNAL1 ZNF468 MAML2 C17orf67 GMPS ANKRD46 NSMCE2 RTKN RAB4B \  0 0 0 0 0 0 0 0 0 0  1 0 0 0 0 0 0 0 0 0  2 0 0 0 0 0 0 0 0 0  3 0 0 0 0 0 0 0 0 0  4 0 0 0 0 0 0 0 0 0  KCNN2 ... PPRC1 EEF1D GNB4 TRADD YAP1 OR4C12 RNF207 UGT8 PTHLH \  0 0 ... 0 0 0 0 0 0 0 0 0  1 0 ... 0 0 0 0 0 0 0 0 0  2 0 ... 0 0 0 0 0 0 0 0 0  3 0 ... 0 0 0 0 0 0 0 0 0  4 0 ... 0 0 0 0 0 0 0 0 0  TRMT61B  0 0  1 0  2 0  3 0  4 0 | [5.1299298e-01, 7.4336469e-01, 2.8196302e-01, 4.0235031e-01,  1.6050115e-01, 0.0000000e+00, 6.1996448e-01, 6.6439432e-01,  0.0000000e+00, 1.2457329e+00, 2.0121148e-01, 6.3954920e-01,  8.8473868e-01, 6.5974343e-01, 1.1049043e+00, 4.3210986e-01,  1.1176988e+00, 1.2880558e+00, 7.4173987e-01, 9.2675954e-01,  1.2931997e-01, 1.0852689e+00, 5.5178863e-01, 6.4118278e-01,  7.0079446e-01, 9.4099391e-01, 1.2364757e+00, 9.8592985e-01,  3.2973191e-01, 0.0000000e+00, 1.5711330e+00, 1.3418105e+00,  1.2515187e-03, 9.8569572e-01, 4.5391452e-01, 1.1224349e+00,  0.0000000e+00, 1.0211439e+00, 1.1171849e+00, 1.1102976e+00,  9.1090041e-01, 0.0000000e+00, 2.3703282e-01, 1.6770387e-01,  1.0728741e+00, 9.9529421e-01, 5.3811502e-01, 7.2175682e-01,  1.3114330e+00, 8.6872017e-01, 1.1113086e+00, 3.7803331e-01,  1.2763109e+00, 1.3927417e+00, 3.4875262e-01, 0.0000000e+00,  9.8515975e-01, 9.5322132e-01, 1.3046898e+00, 9.2364699e-02,  1.5392704e+00, 5.8779502e-01, 5.0042874e-01, 2.8680587e-01,  1.8777006e+00, 1.1496804e+00, 7.0516527e-01, 0.0000000e+00,  1.2728649e-01, 4.5856538e-01, 9.4052577e-01, 1.3986173e-01,  5.3787988e-01, 1.6907090e-01, 4.0179491e-04, 1.9804519e-01,  1.5266418e-01, 9.6847123e-01, 1.3490182e+00, 1.2532742e+00,  5.9600604e-01, 3.2882980e-01, 1.9446510e+00, 5.5090994e-01,  8.6291128e-01, 0.0000000e+00, 0.0000000e+00, 7.0377153e-01,  5.4791719e-01, 0.0000000e+00, 6.8798327e-01, 5.4096663e-01,  3.7651753e-01, 5.0777996e-01, 1.5298971e+00, 6.6808820e-01,  5.2099854e-01, 1.0582457e+00, 5.3636140e-01, 5.4920167e-02] |

# Supplementary Table 4. The ranking of the 100 most important variables using RSF.

| Gene name | Importance | Gene name | Importance | Gene name | Importance | Gene name | Importance |
| --- | --- | --- | --- | --- | --- | --- | --- |
| MAML2 | 1 | LAMA1 | 0.276863504 | GPATCH8 | 0.226202001 | PYGM | 0.198451113 |
| TP53 | 0.745079058 | GRIA4 | 0.275572765 | SLAMF1 | 0.223620523 | DENND4C | 0.197483059 |
| KLHL41 | 0.502097451 | CFAP251 | 0.272991288 | CDHR3 | 0.222329784 | ANKRD11 | 0.19683769 |
| PEG3 | 0.489190061 | RECQL | 0.269119071 | EVPL | 0.221684414 | DSG3 | 0.196515005 |
| DOCK3 | 0.454985479 | MDN1 | 0.266537593 | FGA | 0.221039045 | PACS2 | 0.194901581 |
| ABCA8 | 0.449177154 | TYK2 | 0.2642788 | OR11L1 | 0.219748306 | ADGRF5 | 0.194578896 |
| EPPK1 | 0.408196192 | IFT140 | 0.263956115 | SETDB1 | 0.218457567 | CRAT | 0.193288157 |
| SP3 | 0.407550823 | DYNC2H1 | 0.257825105 | ITSN2 | 0.215876089 | USO1 | 0.193288157 |
| CDKL3 | 0.386576315 | RLF | 0.25750242 | LAMC1 | 0.214262665 | POC1B | 0.192642788 |
| KATNAL1 | 0.363988383 | CADPS | 0.254275573 | ADAMTS20 | 0.213617296 | MDGA2 | 0.191997419 |
| ERBB2 | 0.35559858 | SLITRK4 | 0.249435302 | CPT1A | 0.213294611 | PDZD2 | 0.191674734 |
| NRXN2 | 0.34333656 | CASZ1 | 0.247821878 | AEBP1 | 0.212971926 | MKI67 | 0.19070668 |
| CIT | 0.341077767 | ANK3 | 0.247499193 | COL12A1 | 0.211035818 | SEL1L3 | 0.190383995 |
| ITPR2 | 0.325911584 | UBTF | 0.24588577 | MFSD8 | 0.210713133 | NLRP7 | 0.190383995 |
| ZNF608 | 0.32494353 | GMPS | 0.245563085 | XRN1 | 0.20909971 | BAZ2A | 0.19006131 |
| NELL2 | 0.319135205 | DNAH10 | 0.244917715 | CD68 | 0.205872862 | EEF1AKMT4-ECE2 | 0.189415941 |
| ERBB3 | 0.314294934 | SIN3B | 0.240722814 | NUP98 | 0.204582123 | MYO3B | 0.188447886 |
| SLC9A4 | 0.31332688 | EXOC2 | 0.240077444 | ALMS1 | 0.2029687 | PRR14 | 0.187802517 |
| FSHR | 0.311390771 | IFT88 | 0.237818651 | CNTN3 | 0.2029687 | PARP3 | 0.186511778 |
| GPRASP1 | 0.30719587 | GABRA4 | 0.233946434 | ATP8B3 | 0.2029687 | DNAJC13 | 0.186189093 |
| SLC9A8 | 0.3065505 | DISP3 | 0.230396902 | NLRP9 | 0.201355276 | CPED1 | 0.185866409 |
| IFT52 | 0.297515328 | PRPF3 | 0.228783479 | CHD1L | 0.201355276 | XRCC1 | 0.185221039 |
| GRIP2 | 0.291384318 | AFDN | 0.22749274 | TASOR2 | 0.201032591 | MYO5C | 0.184898354 |
| PARP8 | 0.290093579 | MYH14 | 0.22749274 | ATP7B | 0.199419167 | CNGA2 | 0.184252985 |
| HIP1 | 0.285898677 | TANC1 | 0.22684737 | RUFY1 | 0.199096483 | KRT9 | 0.184252985 |

# Supplementary Table 5. The influence of randomness of neural network training on results.

| Times | MCE | Binary accuracy | Features | C-index | R square |
| --- | --- | --- | --- | --- | --- |
| 1 | 0.0006 | 1 | 25 | 0.877 | 0.329 |
| 2 | 0.0006 | 1 | 21 | 0.865 | 0.315 |
| 3 | 0.0006 | 1 | 27 | 0.905 | 0.394 |
| 4 | 0.0005 | 1 | 17 | 0.87 | 0.297 |
| 5 | 0.0006 | 1 | 22 | 0.885 | 0.349 |
| 6 | 0.0006 | 1 | 44 | 0.915 | 0.425 |
| 7 | 0.0006 | 1 | 29 | 0.899 | 0.393 |
| 8 | 0.0006 | 1 | 30 | 0.888 | 0.355 |
| 9 | 0.0006 | 1 | 48 | 0.895 | 0.415 |
| 10 | 0.0006 | 1 | 37 | 0.897 | 0.439 |

Note: We retrained the auto-encoder 10 times to see the effect of randomness on the results.


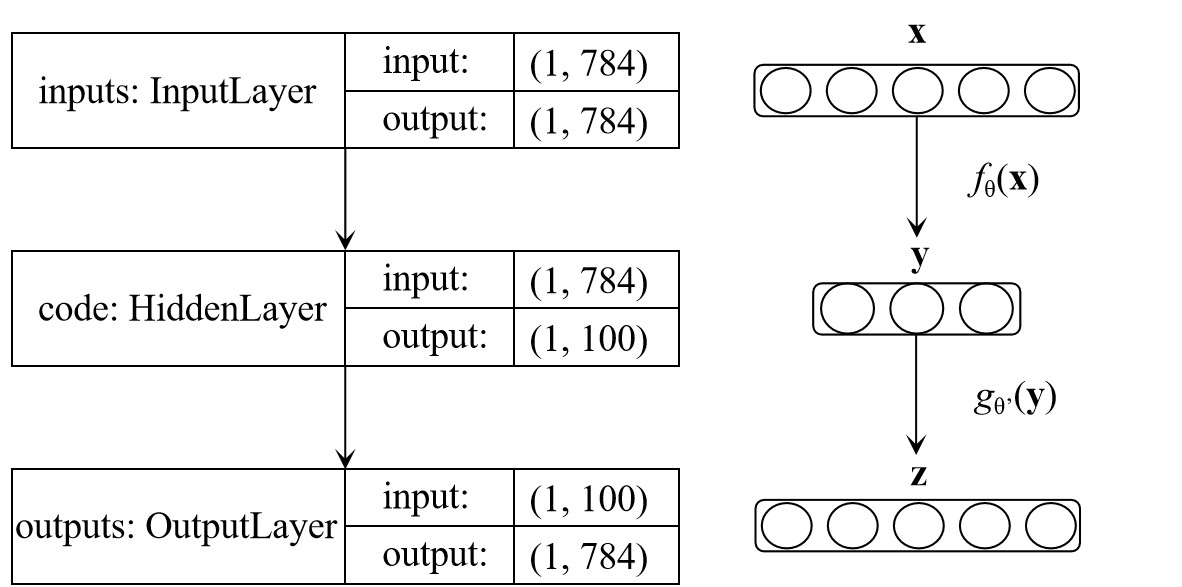


# Supplement Figure 1. The construction of the auto-encoder in MNIST data


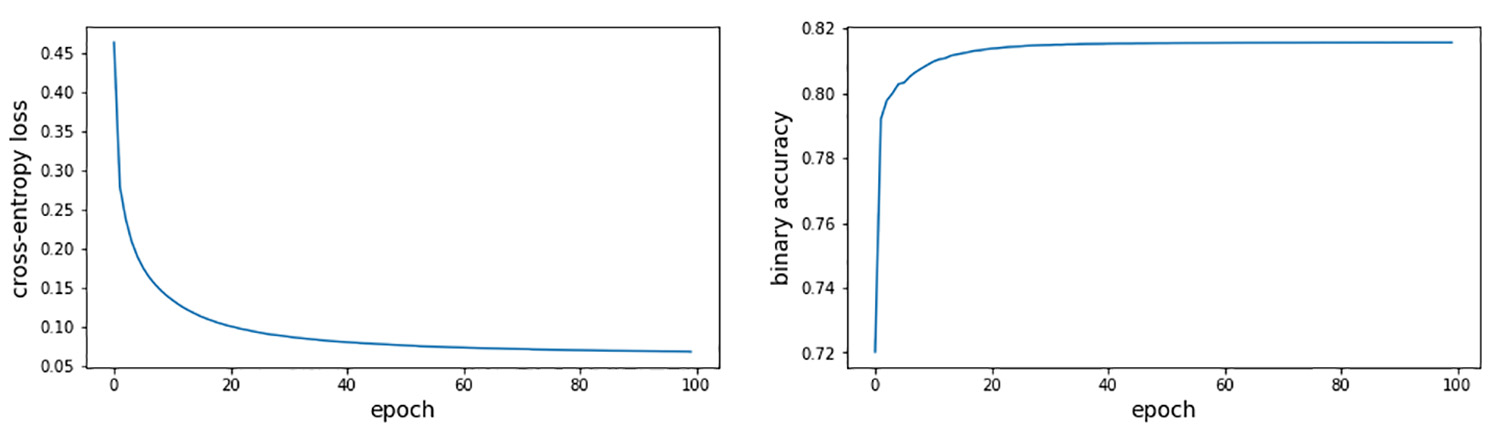


A


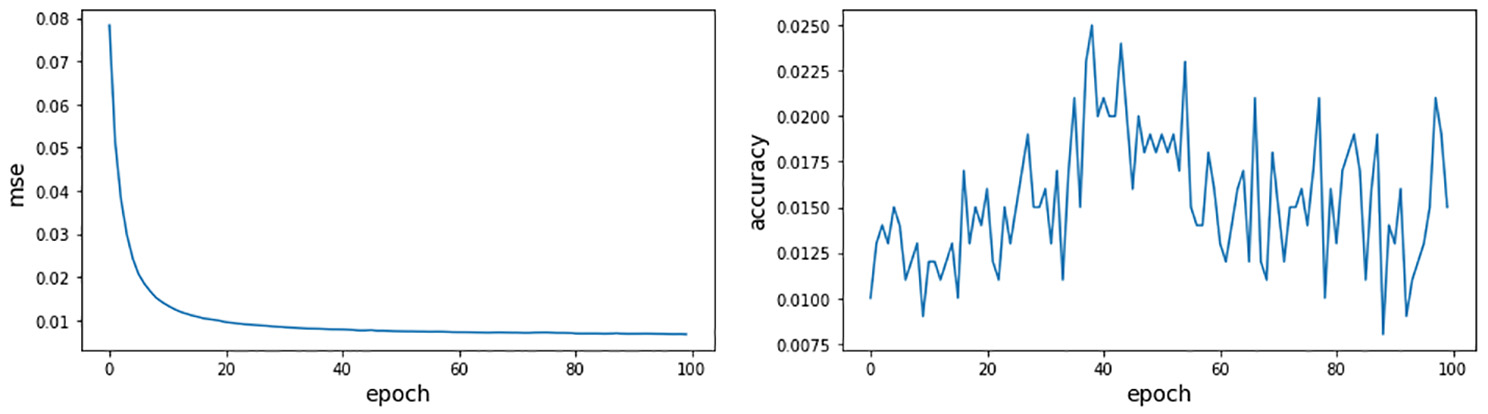


B

# Supplement Figure 2. Loss function value and accuracy of the auto-encoder in MNIST training data by the epoch times. (A) Using sigmoid function (B) Using ReLU function


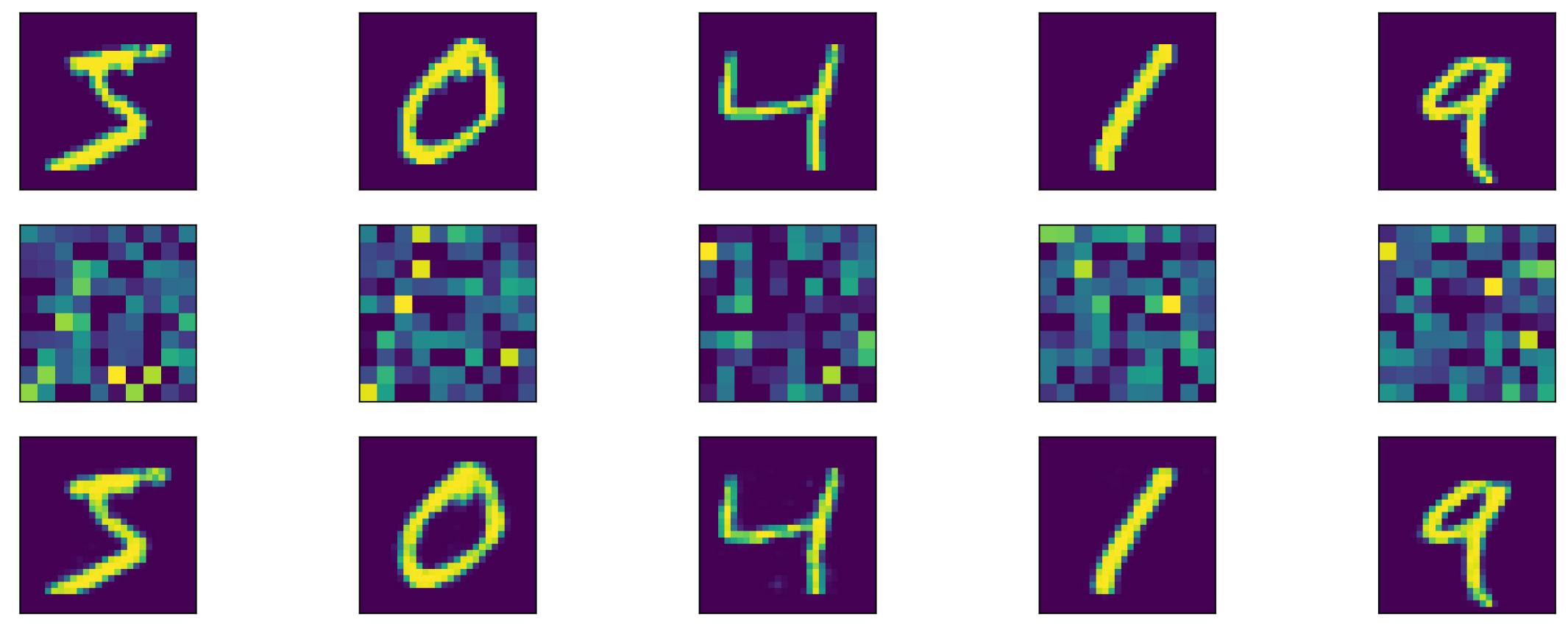


A


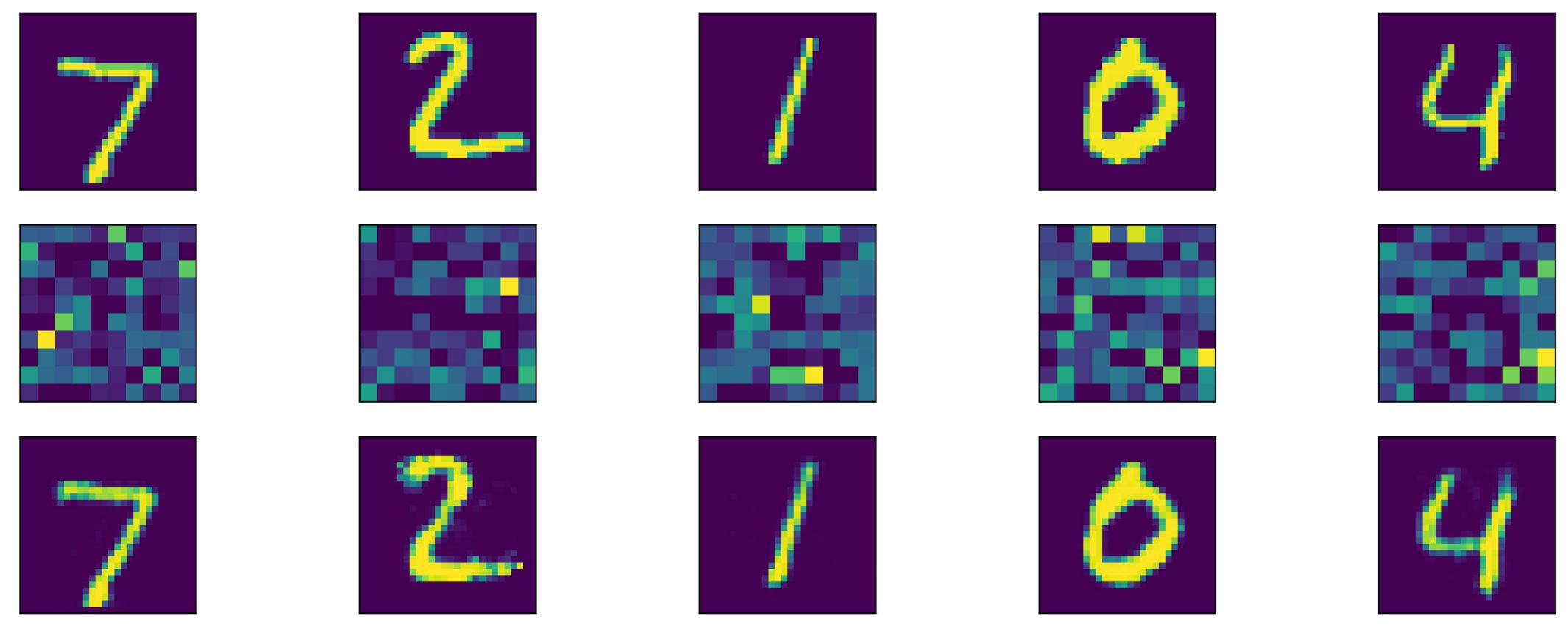


B


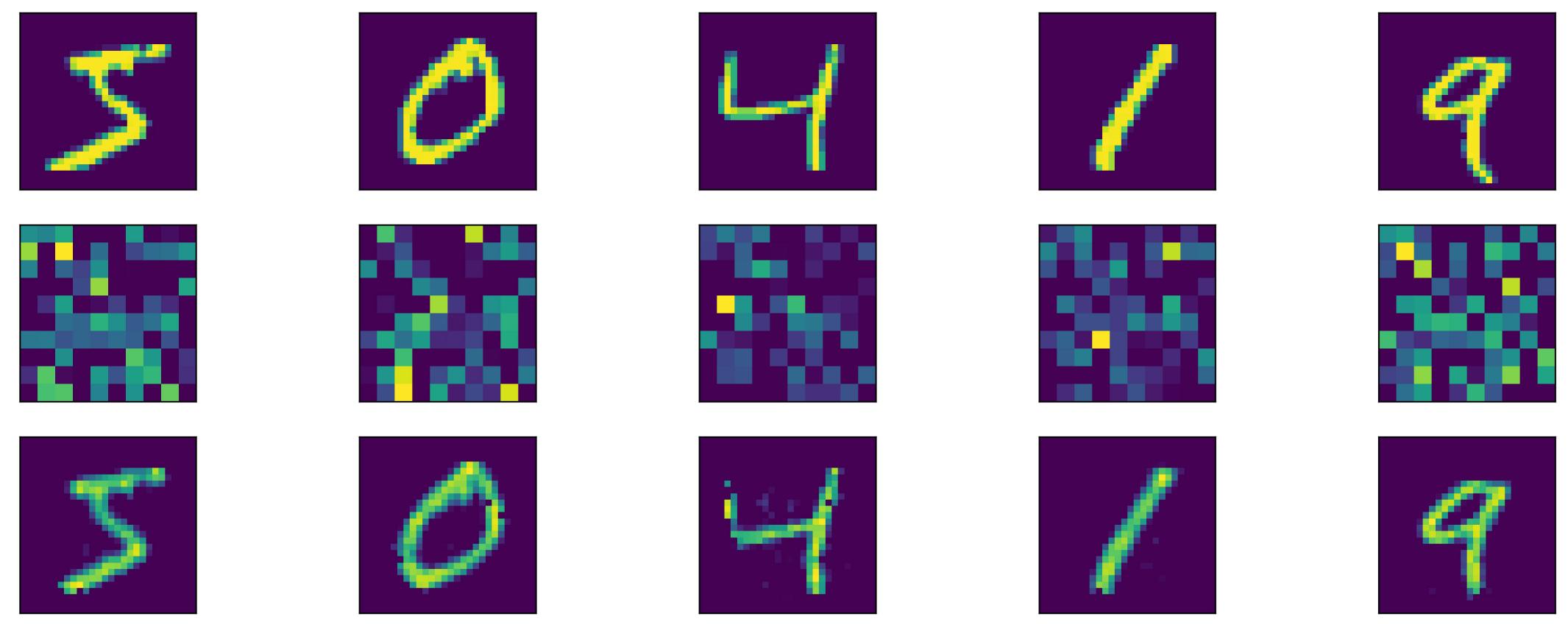


C


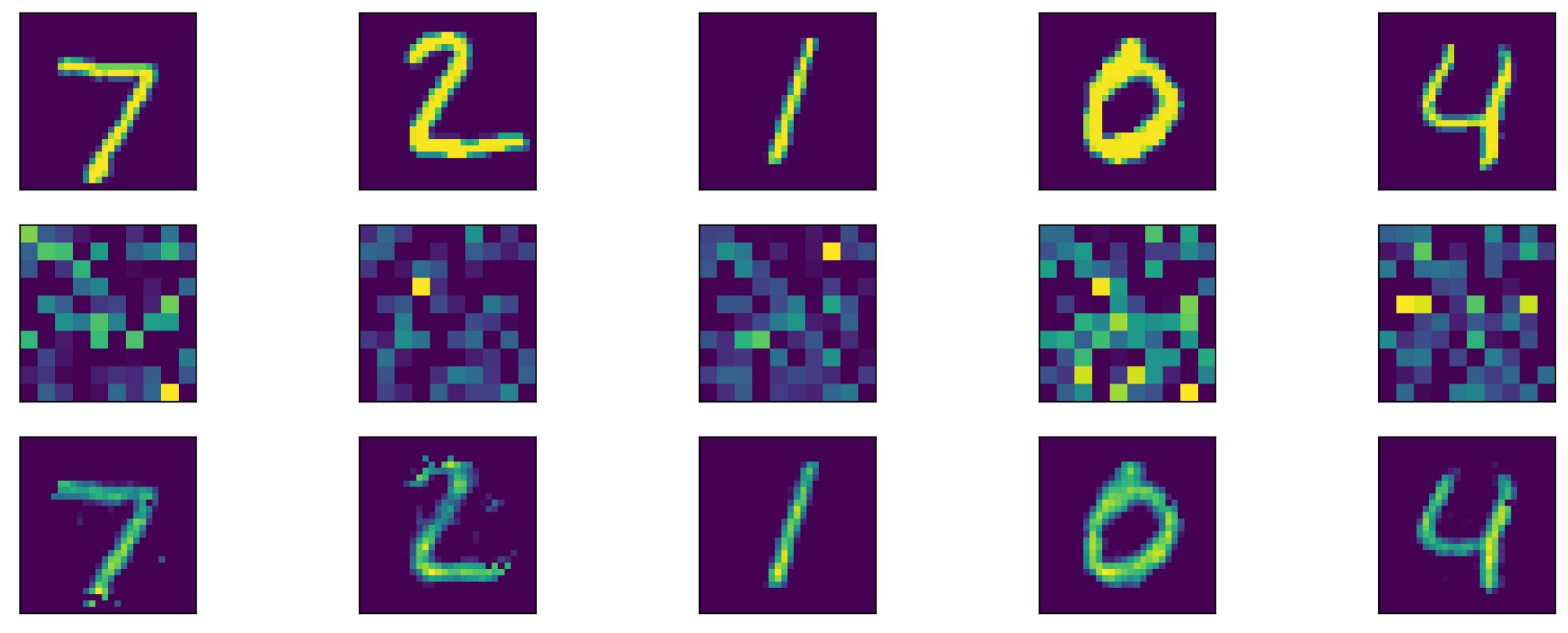


D

# Supplement Figure 3. The first five image of MNIST training data and testing data. (A) Training data using sigmoid function (B) Testing data using sigmoid function (C) Training data using ReLU function (D) Testing data using ReLU function


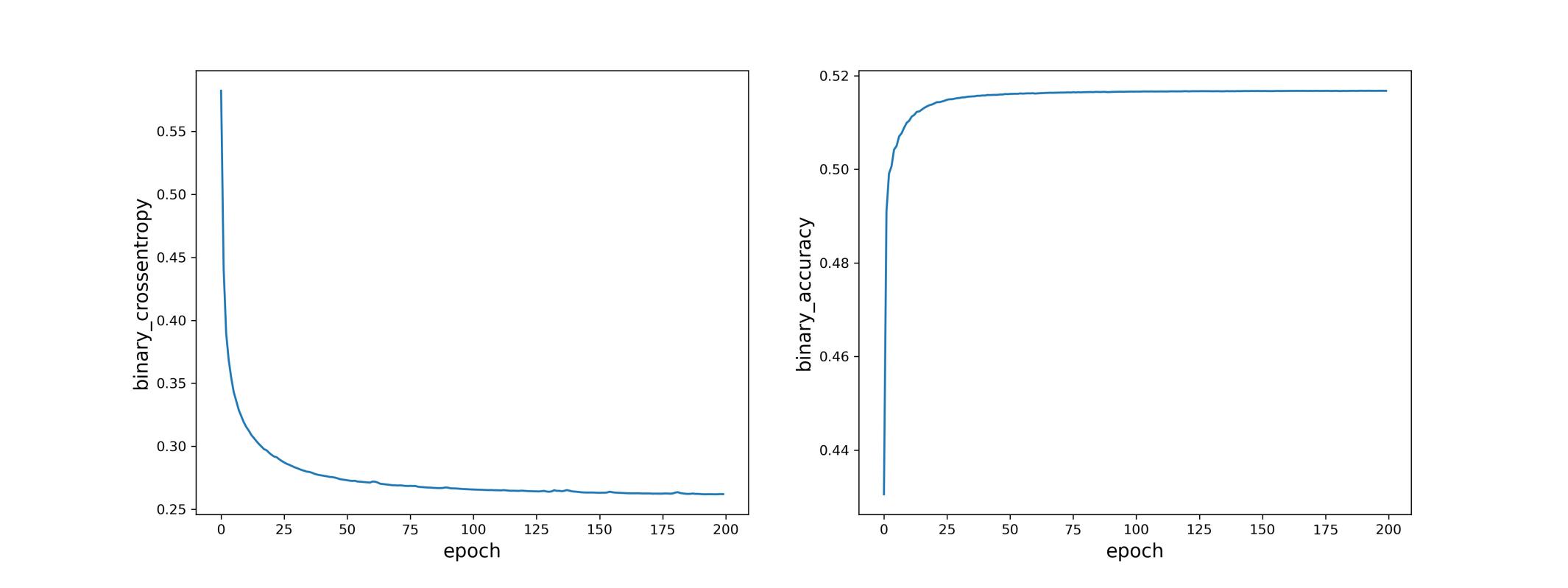


A


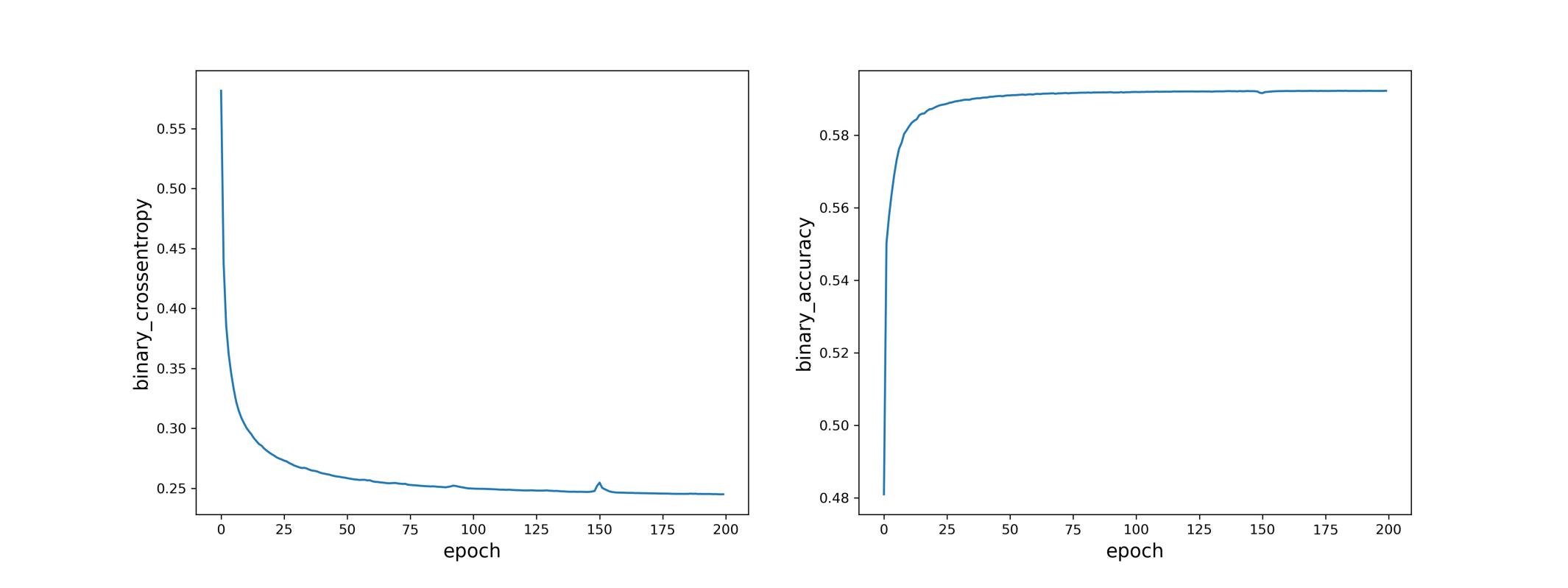


B

# Supplement Figure 4. Loss function value and accuracy of the auto-encoder in fashion MNIST training data by the epoch times. (A) Original data using sigmoid function (B) Corrupted data using sigmoid function


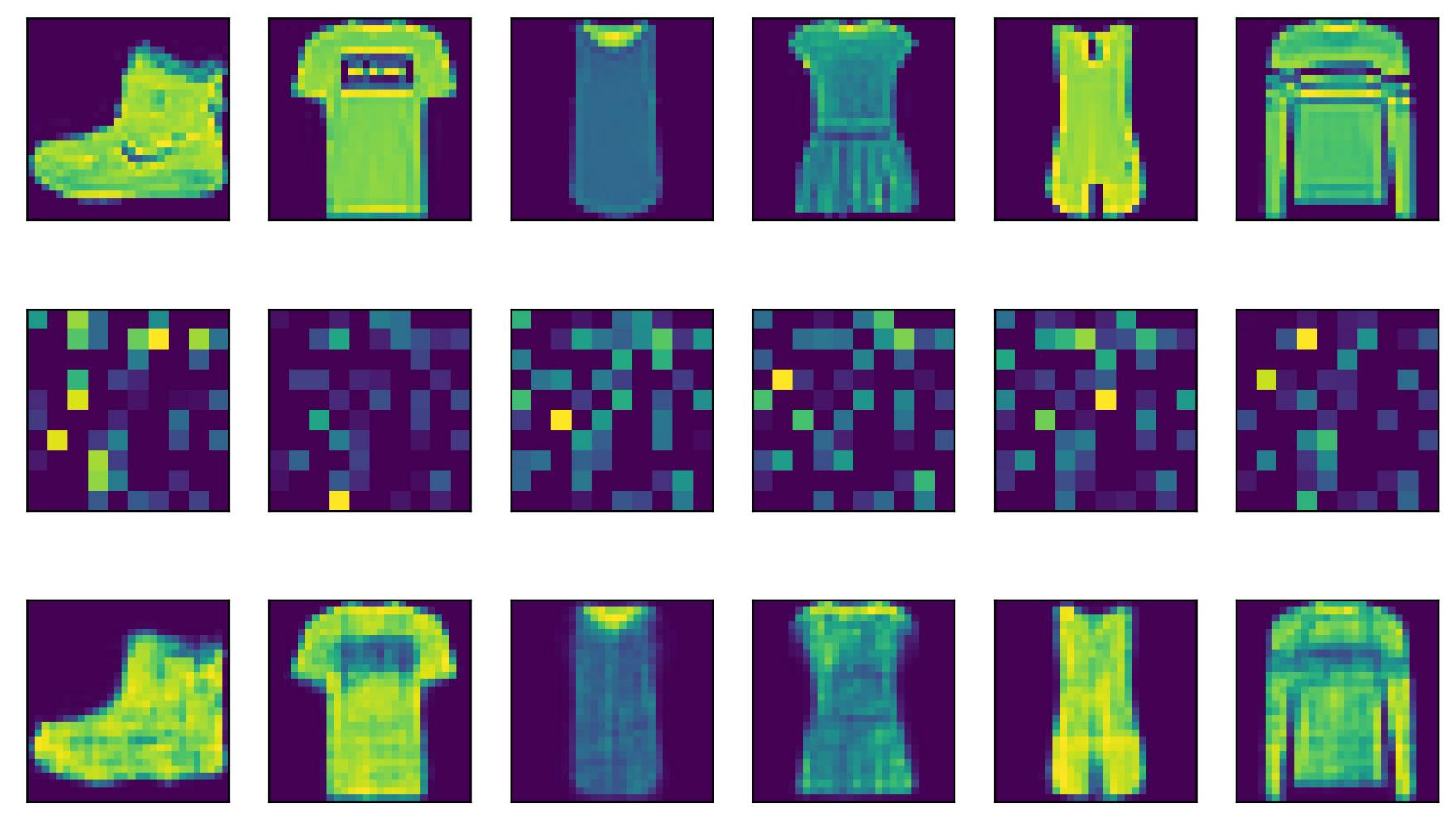


A


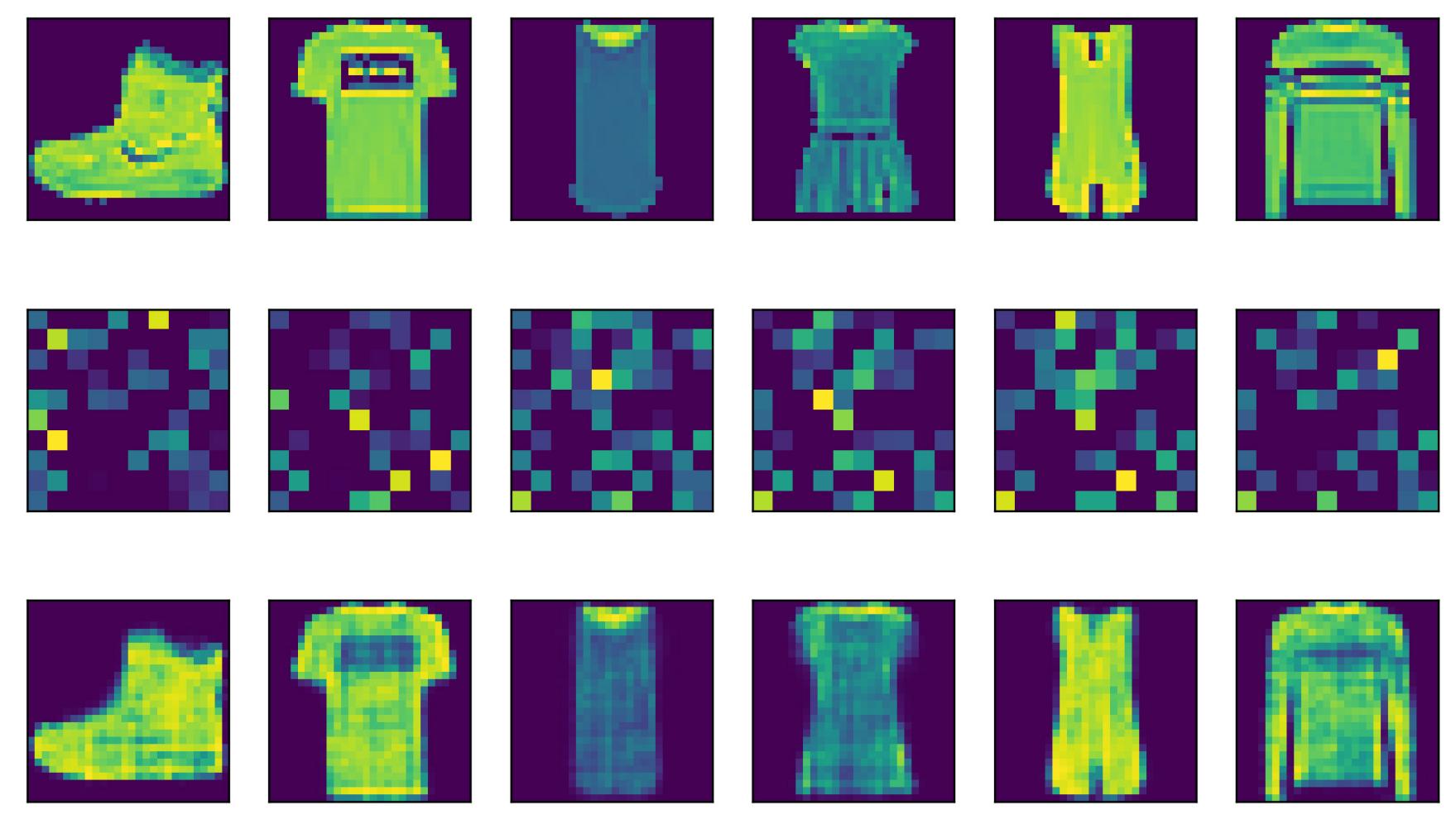


B


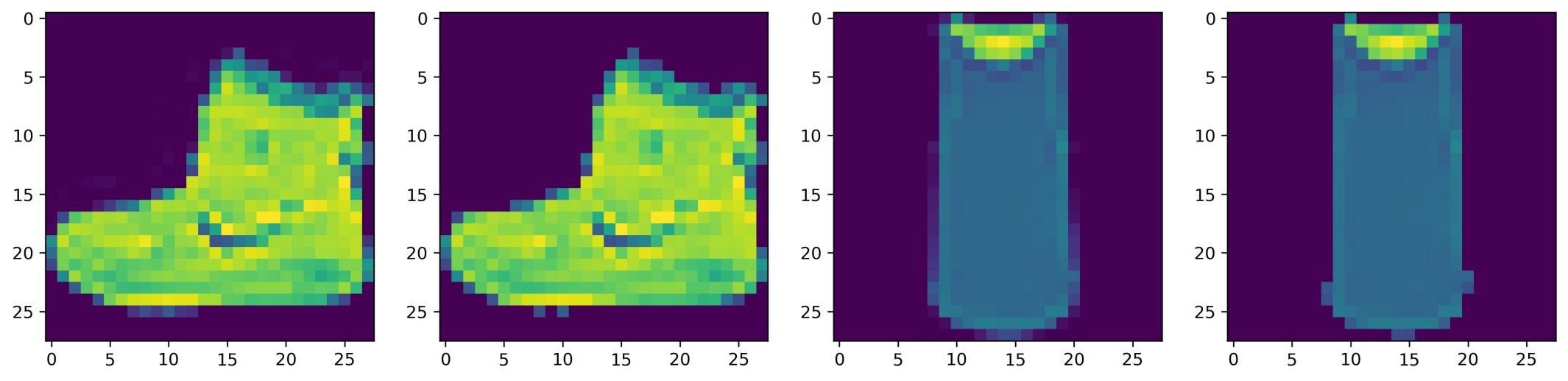


C

# Supplementary Figure 5. The first five image of fashion MNIST training data. (A) Original data using sigmoid function (B) Corrupted data using sigmoid function (C) Images of original data V.S. corrupted data. The first and third images were original data, the second and fourth images were corrupted data


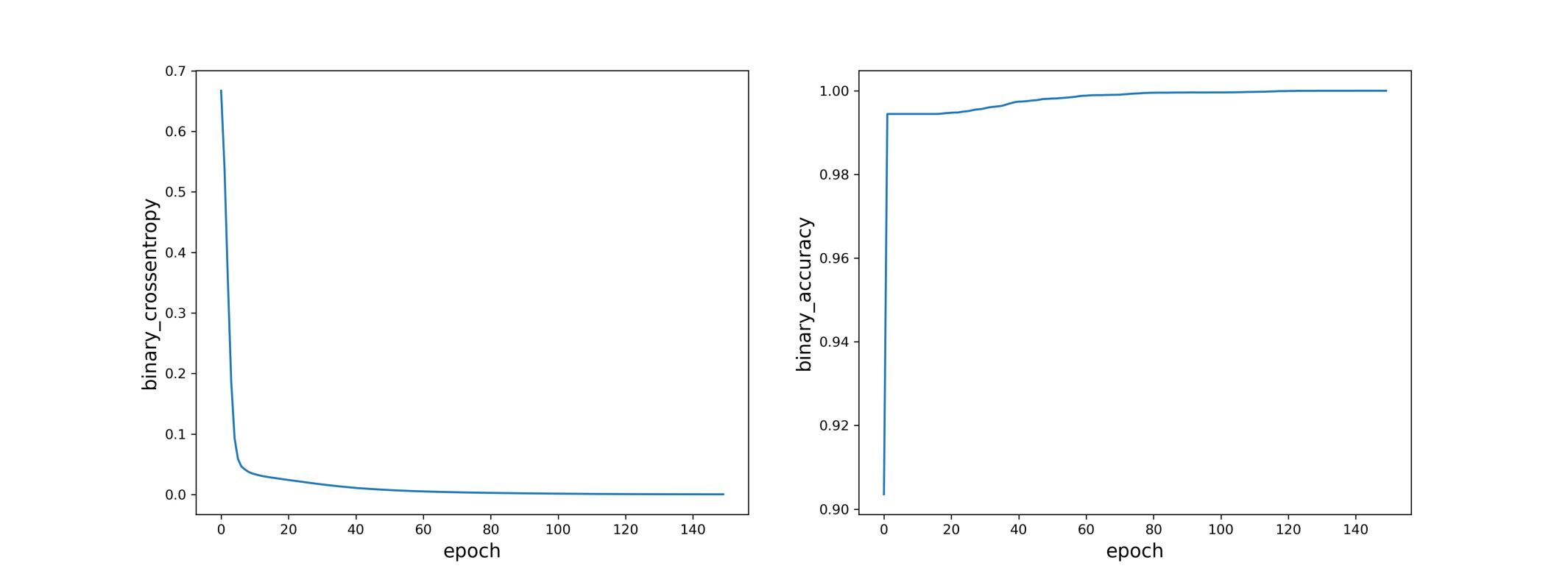


A


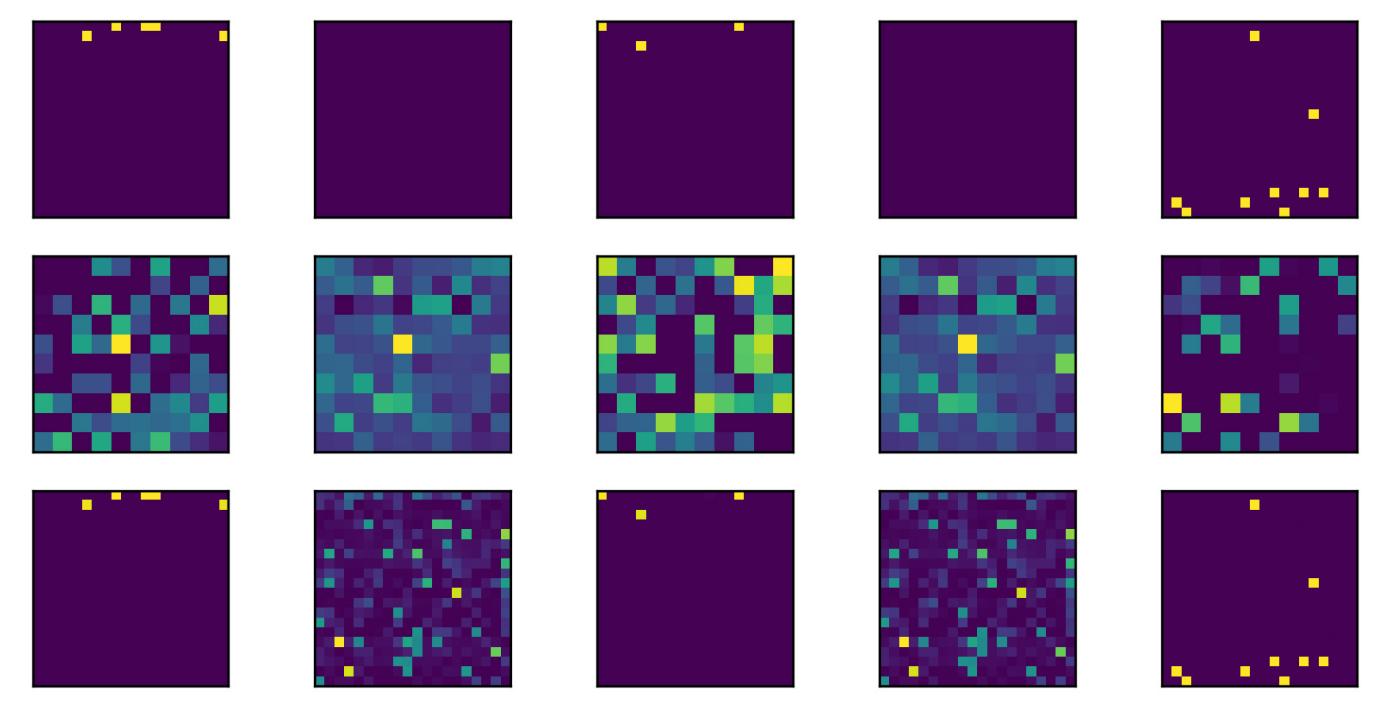


B

# Supplementary Figure 6. Auto-encoder feature selection for highly sparse binary predictors. (A) Loss function value and accuracy of the auto-encoder in simulated data by the epoch times (B) The first five visualized genetic signal of simulated data


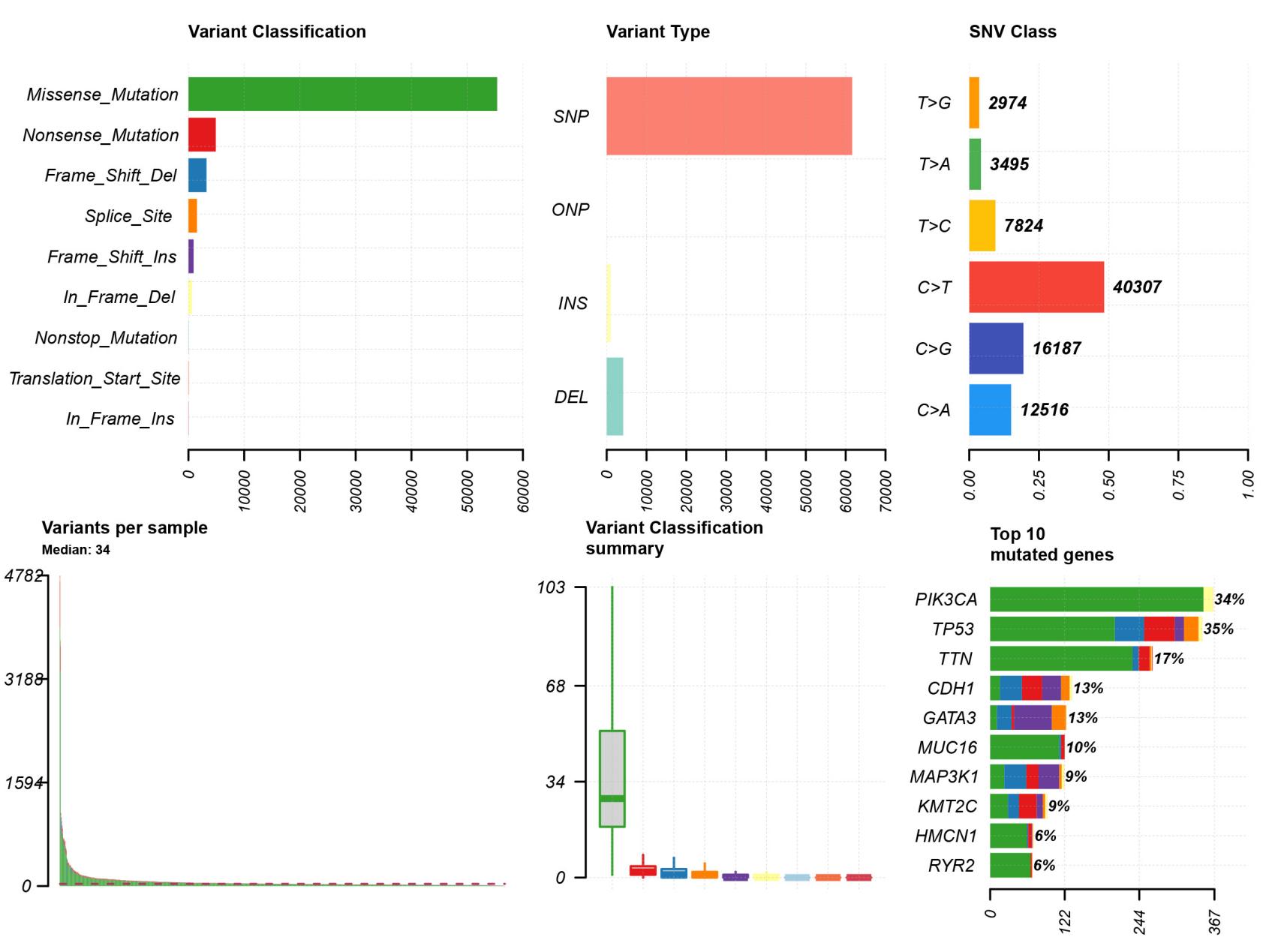


A


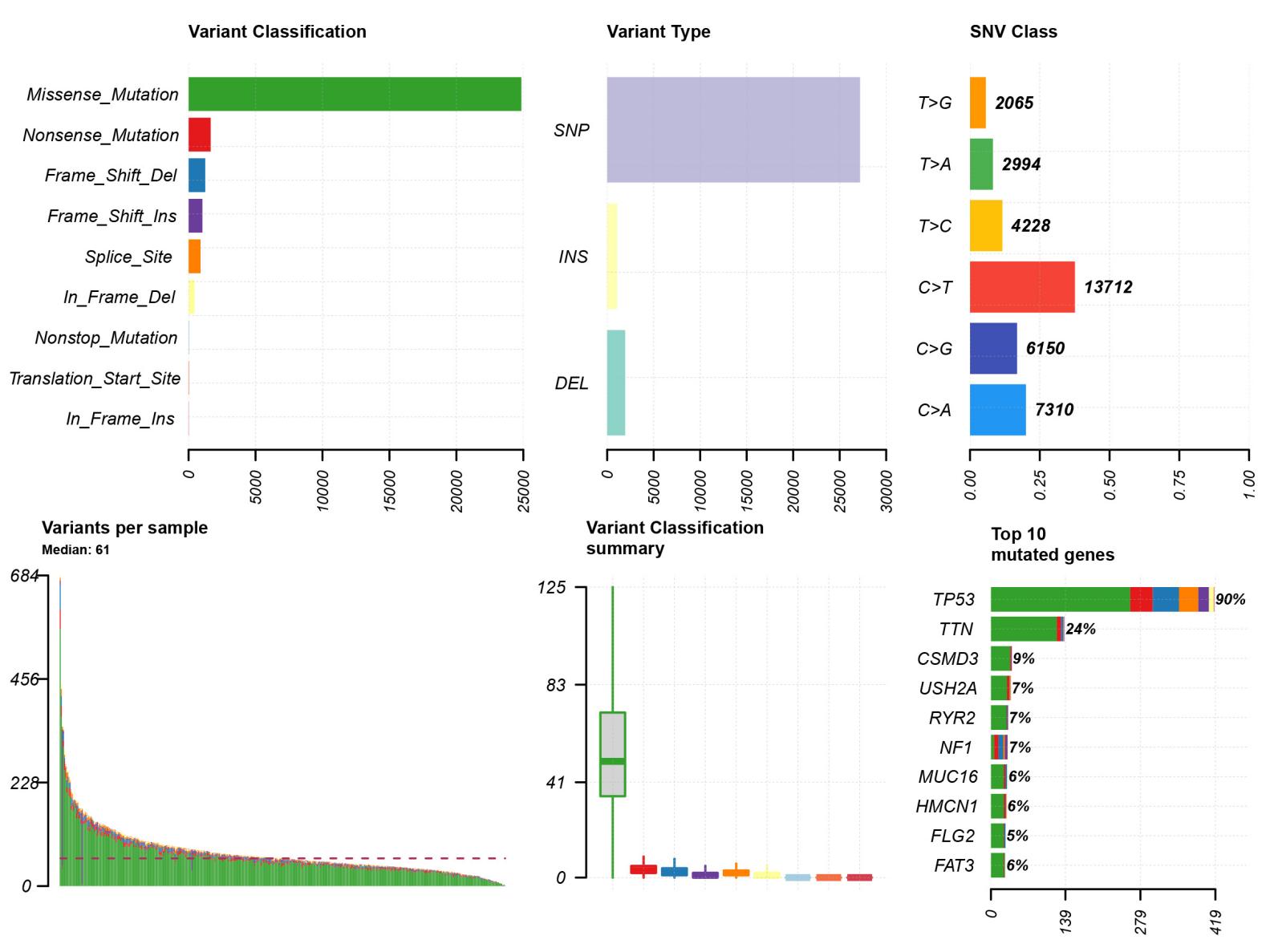


B

# Supplementary Figure 7. The summary of SNVs in BRCA data and OV data. (A) BRCA data (B) OV data


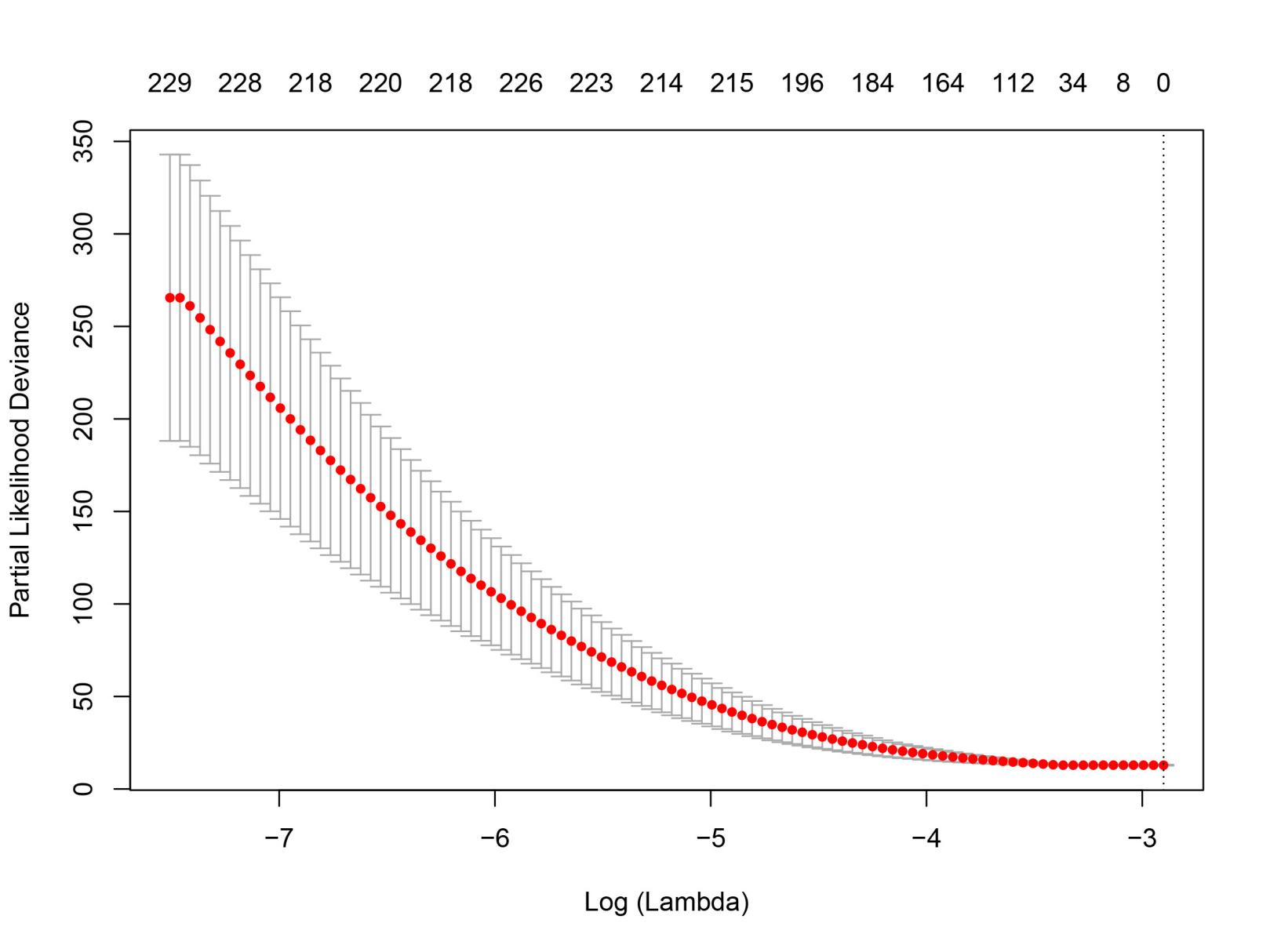


# Supplementary Figure 8. The process of the LASSO to directly select predictors using 1,936 genotype data in BRCA.


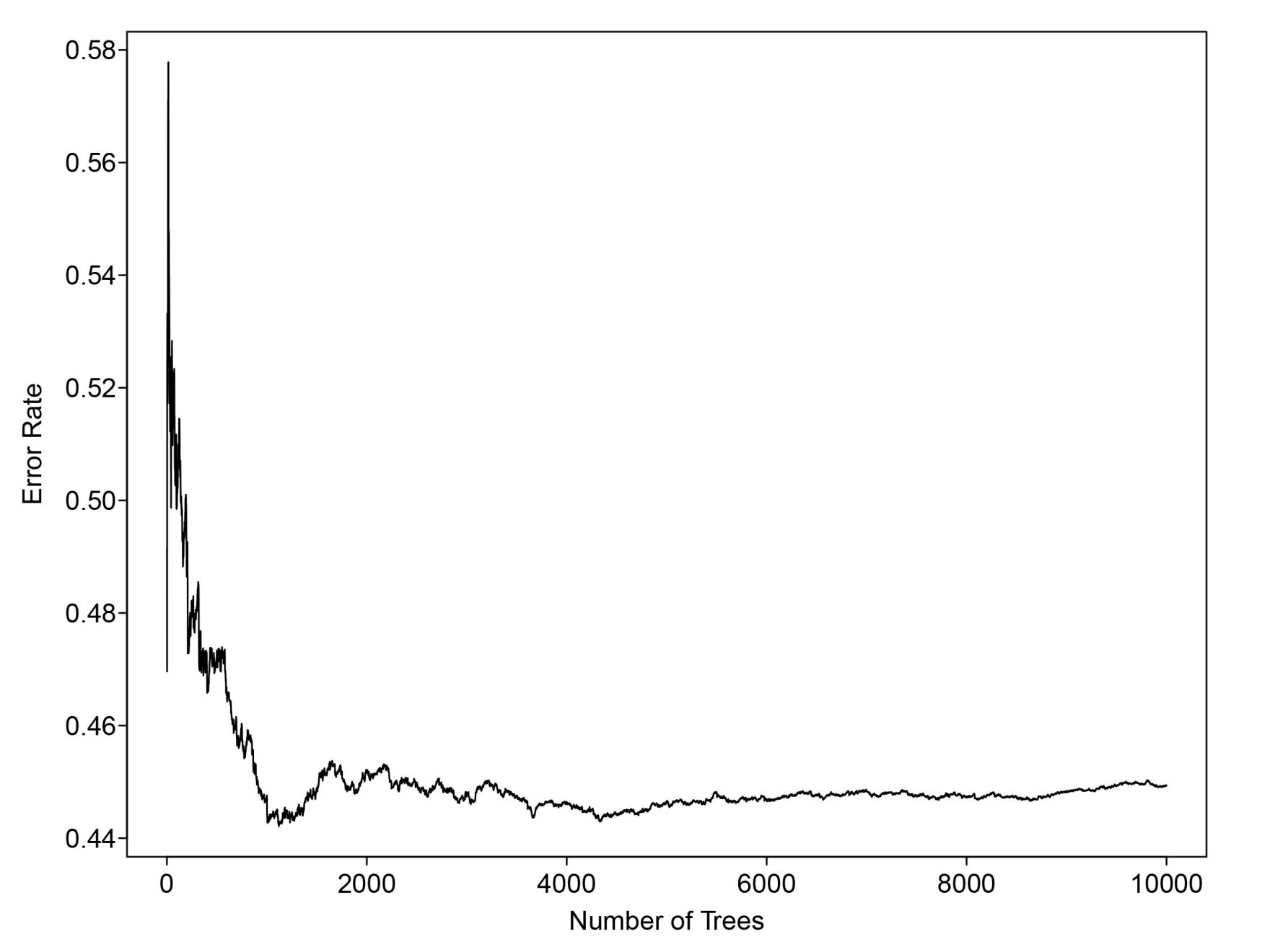


A


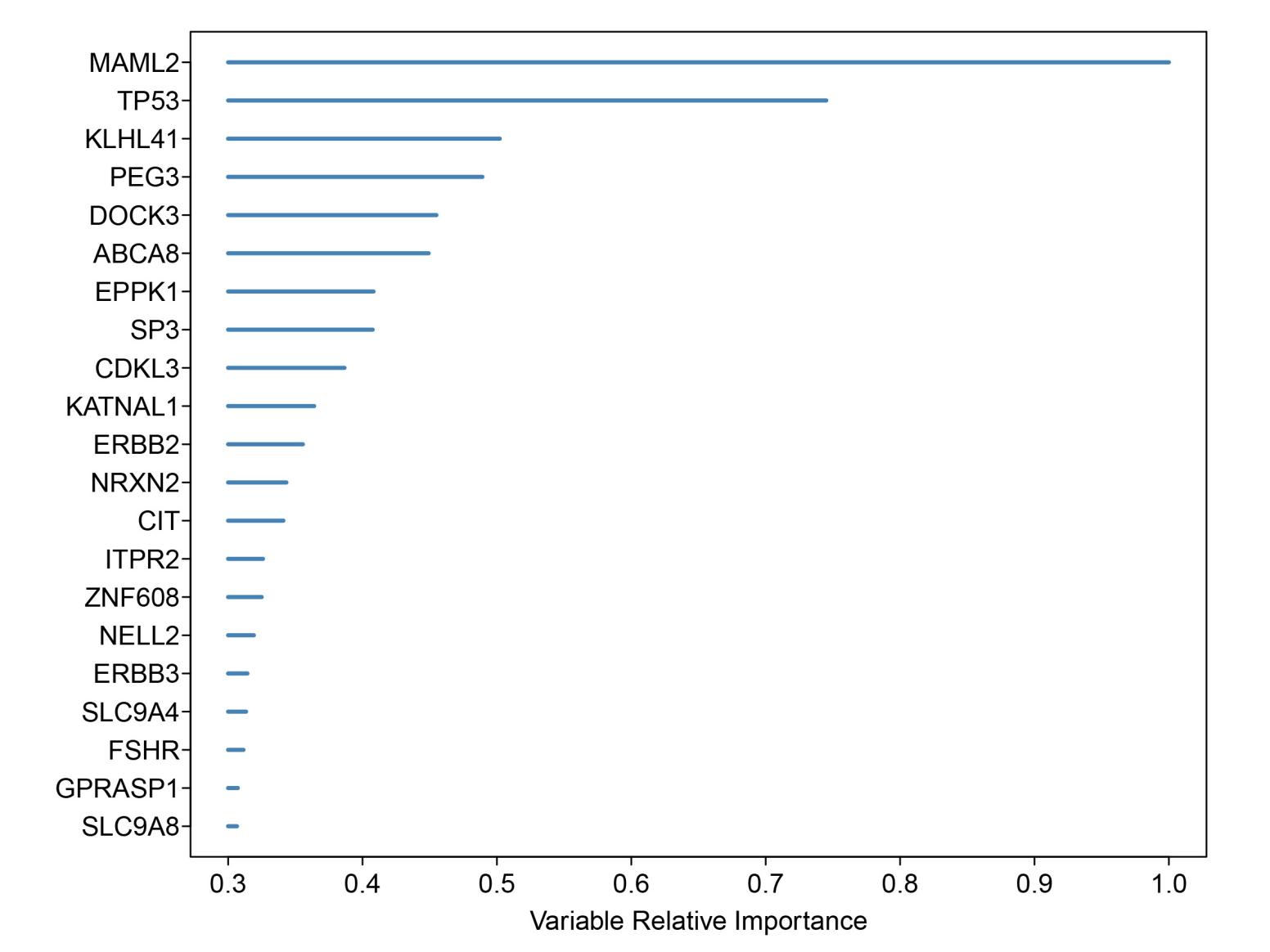


B

# Supplementary Figure 9. The process of variables selection using RSF. (A) Error rate by number of trees (B) 21 variables with importance index greater than 0.3


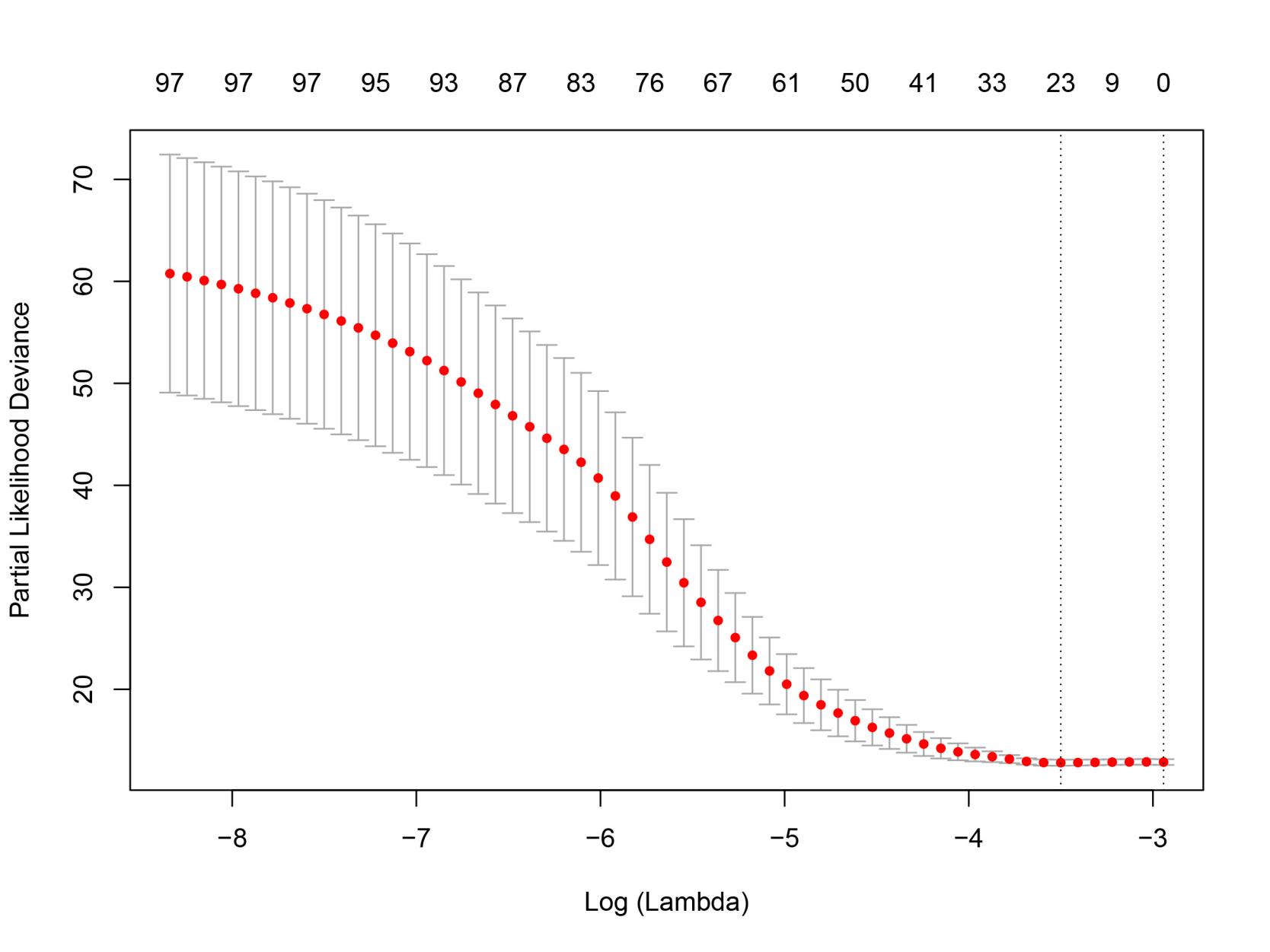


# Supplementary Figure 10. The process of the LASSO to select predictors using 100 most important variables selected using RSF in BRCA.


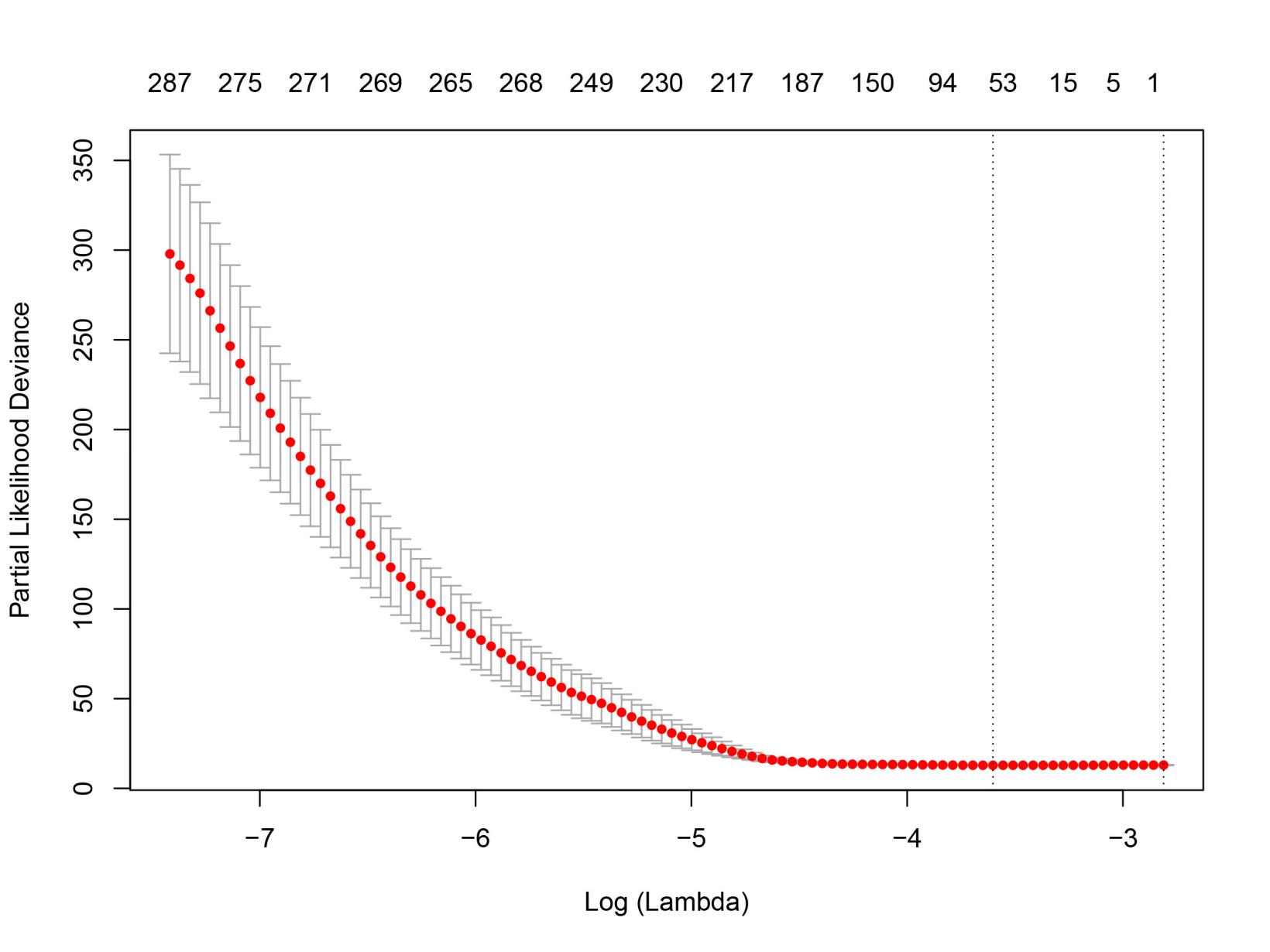


# Supplementary Figure 11. The process of the LASSO to directly select predictors using 1,936 gene expression data in BRCA.


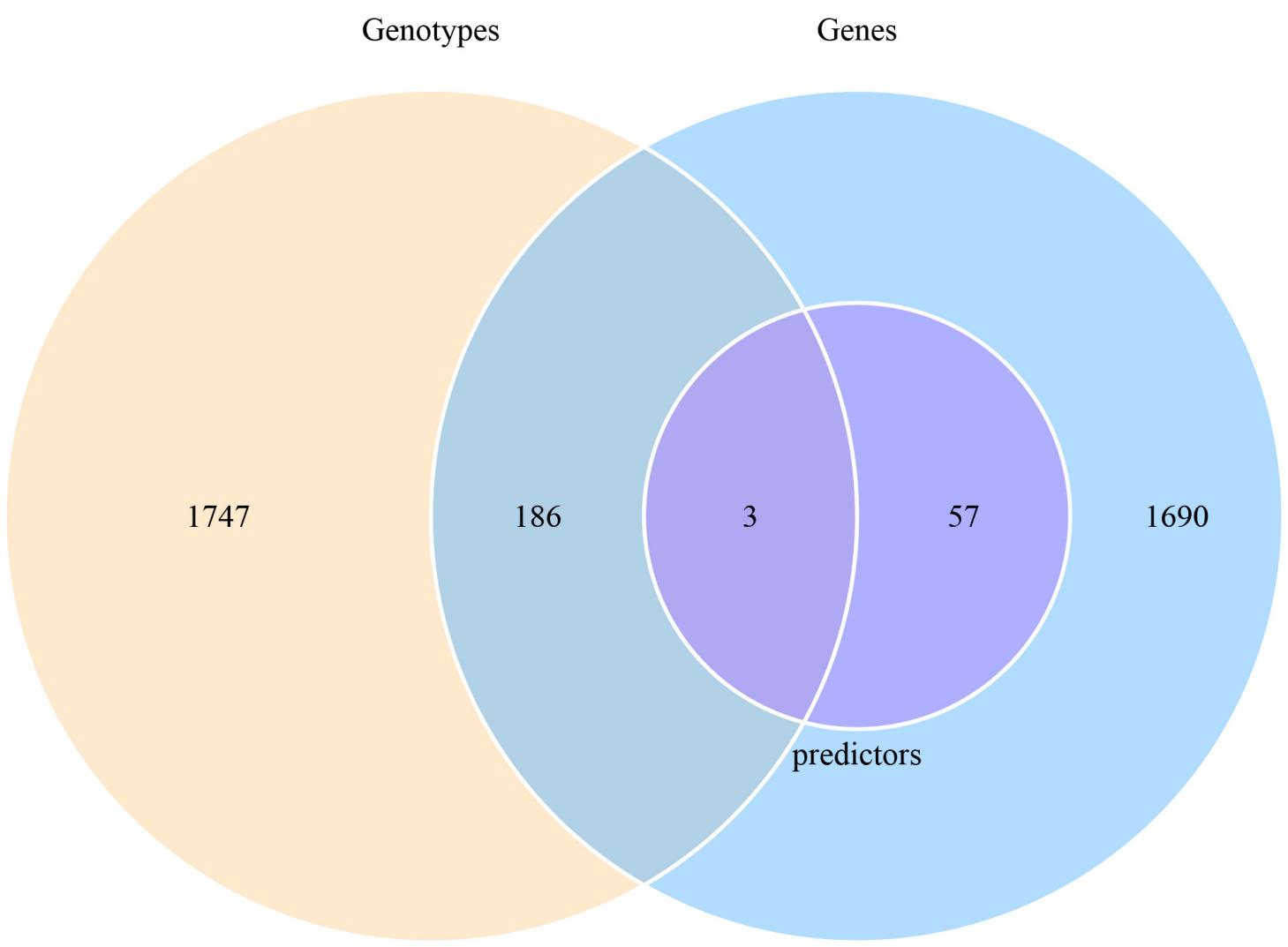


# Supplementary Figure 12. The Venn plot about 1,936 genotype, 1,936 genes and 60 predictors


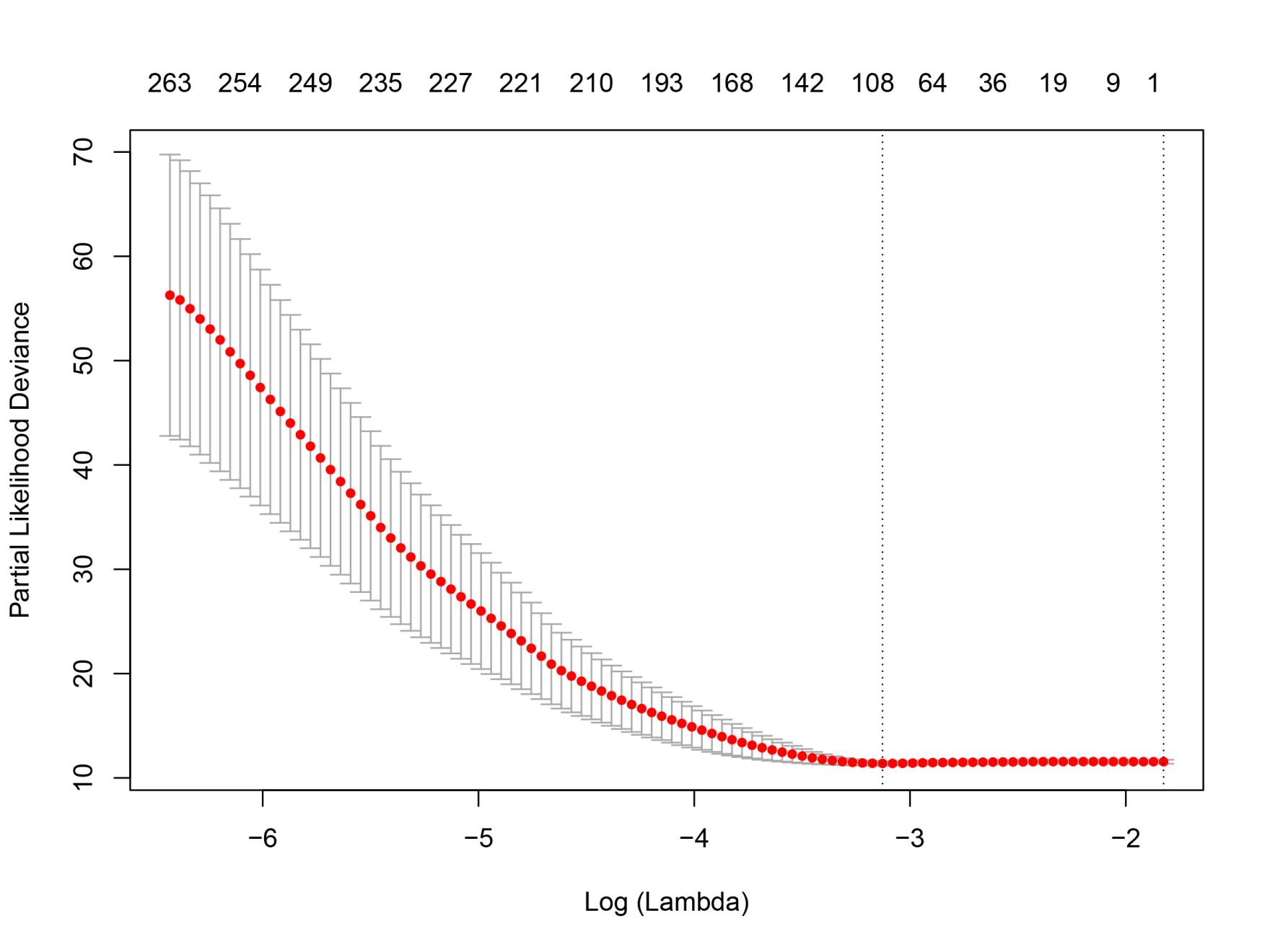


A


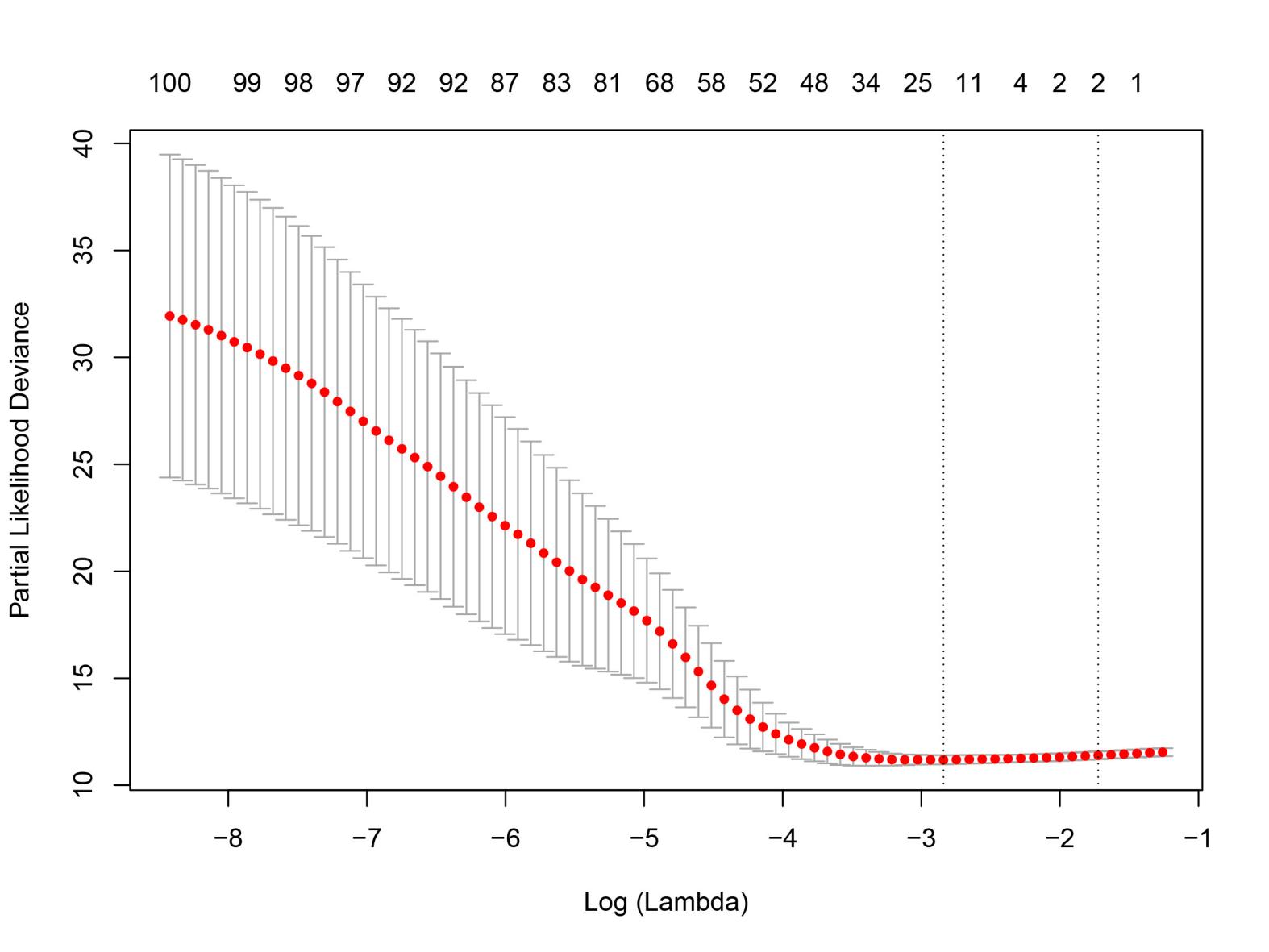


B

# Supplementary Figure 13. The process of the LASSO to select predictors using genotype data in OV. (A) Directly select predictors using 1,089 genotype data. (B) 19 features were selected from 100 extracted features using the LASSO process.

*The LASSO selection* *for highly sparse binary predictors*

We set up five scenarios. For each scenario, we generated n (= 200) observations, each subject i with a survival response, consisting of an observed censored survival time t^(i)^ and a censoring indicator d^(i)^, and a vector of **m** (= 15, 100, 200, 300, 400) binary predictors **x**^(i)^ = (x^(i)^_1_, . . . , x^(i)^_m_). In particular, we used R package *bhGLM* (Yi, Tang et al. 2019) (functions “sim.x()” and “sim.y()” are used to generate different types of high dimension variables and responses, and specify the correlation between covariates within and between groups) to generate the simulated survival responses and genotype predictors. The vector **x**^(i)^ was generated with 50 elements in a group, where the intra-group correlation was set to 0.6 and the inter-group correlation was 0. Detailed process referenced to (Tang, Shen et al. 2017). Specially, with a genotype predictor an individual was coded 1 if a rare allele was present and 0 otherwise. Thus the genotype predictors were binary.

We set fifteen coefficients β_1_ to β_15_ as non-zero, six of which were negative. The rest of other coefficients were set to be zero. The preset 15 non-zero coefficient values for five simulation scenarios were -0.7786371, 1.1011591, 1.5572742, -1.9072635, 2.0600801, -0.7786371, 1.1011591, 1.5572742, -1.9072635, 2.0600801, -0.7786371, 1.1011591, 1.5572742, -1.9072635, 2.0600801, respectively. We set the mutation frequency of these 15 genotype predictors to 0.01, and the rest of others to 0.002. Thus the overall proportion of zero is more than 99%. We analyzed each simulated scenarios using the LASSO Cox model with penalty parameter tuning conducted by 10-fold cross-validation that was implemented in the R package *glmnet* (Engebretsen and Bohlin 2019) for replication with 100 times and recorded average numbers of non-zero predictors that were caught by the LASSO. The result is shown in **Table S1**. As noise variables increase, power of the LASSO to selecting non-zero coefficients plummeted (from 10.83 to 2.96) and it was prone to select more zero coefficient variables. In addition, the possibility that the LASSO would not be able to pick any predictors increases (from 0.02 to 0.23).

**Reference**

Engebretsen, S. and J. Bohlin (2019). "Statistical predictions with glmnet." Clin Epigenetics **11**(1): 123.

Tang, Z., Y. Shen, X. Zhang and N. Yi (2017). "The spike-and-slab lasso Cox model for survival prediction and associated genes detection." Bioinformatics **33**(18): 2799-2807.

Yi, N. J., Z. X. Tang, X. Y. Zhang and B. Y. Guo (2019). "BhGLM: Bayesian hierarchical GLMs and survival models, with applications to genomics and epidemiology." Bioinformatics **35**(8): 1419-1421.
